# Using synthetic chromosome controls to evaluate the sequencing of difficult regions within the human genome

**DOI:** 10.1186/s13059-021-02579-6

**Published:** 2022-01-12

**Authors:** Andre L. M. Reis, Ira W. Deveson, Bindu Swapna Madala, Ted Wong, Chris Barker, Joshua Xu, Niall Lennon, Weida Tong, Tim R. Mercer, on behalf of the SEQC2 Consortium

**Affiliations:** 1 Kinghorn Centre for Clinical Genomics, Garvan Institute of Medical Research, Sydney, NSW Australia; 2 St Vincent’s Clinical School, University of New South Wales, Sydney, NSW Australia; 3 Genomics and Epigenetics Theme, Garvan Institute of Medical Research, Sydney, NSW Australia; 4 Division of Bioinformatics and Biostatistics, National Center for Toxicological Research, U.S. Food and Drug Administration, Jefferson, AR 72079 USA; 5 Broad Institute of MIT and Harvard, Cambridge, MA 02142 USA; 6 Australian Institute for Biotechnology and Nanoengineering, University of Queensland, Brisbane, QLD Australia

## Abstract

**Background:**

Next-generation sequencing (NGS) can identify mutations in the human genome that cause disease and has been widely adopted in clinical diagnosis. However, the human genome contains many polymorphic, low-complexity, and repetitive regions that are difficult to sequence and analyze. Despite their difficulty, these regions include many clinically important sequences that can inform the treatment of human diseases and improve the diagnostic yield of NGS.

**Results:**

To evaluate the accuracy by which these difficult regions are analyzed with NGS, we built an in silico decoy chromosome, along with corresponding synthetic DNA reference controls, that encode difficult and clinically important human genome regions, including repeats, microsatellites, HLA genes, and immune receptors. These controls provide a known ground-truth reference against which to measure the performance of diverse sequencing technologies, reagents, and bioinformatic tools. Using this approach, we provide a comprehensive evaluation of short- and long-read sequencing instruments, library preparation methods, and software tools and identify the errors and systematic bias that confound our resolution of these remaining difficult regions.

**Conclusions:**

This study provides an analytical validation of diagnosis using NGS in difficult regions of the human genome and highlights the challenges that remain to resolve these difficult regions.

**Supplementary Information:**

The online version contains supplementary material available at 10.1186/s13059-021-02579-6.

## Background

Informed patient care requires the accurate diagnosis of the genetic alterations that cause inherited diseases and cancer. Next-generation sequencing (NGS) can identify these mutations within a patient genome and has been widely adopted in clinical practice. However, NGS suffers from errors and biases that can confound clinical interpretation and diagnosis [1–3].

The size, complexity, and repetitiveness of the human genome can cause sequencing errors and ambiguous alignments, which confound the analysis and interpretation of variants at these difficult sites [4]. In the absence of a solution, clinical NGS is currently limited to “easy” regions of the genome and simple mutation types where error rates are sufficiently low, while “difficult” regions or complex mutations are largely ignored, resulting in a lower diagnostic yield for NGS.

The human genome sequence contains many difficult, polymorphic, and repetitive regions that remain a challenge for NGS. These difficult regions present a challenge to sequencing, alignment, and bioinformatic analysis [5–7]. However, some genetic sequences with clinical importance reside in such difficult regions, including polymorphic loci, such as *HLA* genes, low-complexity sequences such as microsatellites, and complex loci such as immune receptors [8]. Given their clinical importance, it is critical to understand and improve the accuracy and precision by which these genetic features can be resolved using NGS.

Well-characterized reference genome materials provide a ground-truth reference or factual data for evaluating the performance of NGS [9]. Efforts like the 1000 Genomes Project and the Genome in a Bottle Consortium (GIAB) have provided well-characterized genomes, with high-confidence annotation of genetic variants that have been widely adopted by the genomics community [10–12]. However, despite their advantages, these genomes often do not include pathogenic variants, and characterization of difficult regions within these genomes still remains limited using current sequencing technologies.

Recently, the US FDA-led Sequencing Quality Control Phase 2 (SEQC2) Consortium conducted a broad range of projects to interrogate the technical reliability and clinical utility of NGS in cancer genomics, liquid biopsy, single-cell sequencing, and DNA- and RNA-seq [13]. A primary objective of this initiative is to establish working standards and reference controls for NGS, including in difficult human genome regions that are refractory to analysis with NGS, and which are under-represented in current reference materials. Accordingly, as part of this initiative, we developed synthetic controls to address this shortcoming, and evaluate these difficult, yet clinically important regions of the human genome.

Synthetic controls are an alternative approach to providing ground-truth reference materials. While they do not recapitulate the full size and scope of genome materials, they can provide an unambiguous representation of difficult genome regions. Recently, we have developed synthetic DNA spike-in controls (termed *sequins*) that faithfully emulate features of the human genome, such as genetic variation. Sequins represent mirror images of naturally occurring DNA present in the NA12878 reference genome sample, but because of DNA’s 5′-3′ directionality, they are entirely distinguishable from their natural counterparts (see the “Methods” section [14];). These controls can be added to a DNA sample prior to library preparation and undergo concurrent sequencing and are exposed to the same technical biases of the accompanying sample [14–16]. Given the full sequence of sequins is known, they can unambiguously represent difficult sequences with high confidence. This is particularly important given these difficult regions are highly polymorphic due to the challenge they pose to DNA replication. Accordingly, sequins provide an ideal ground truth by which to evaluate the performance of sequencing experiments and technologies.

**Fig. 1:**
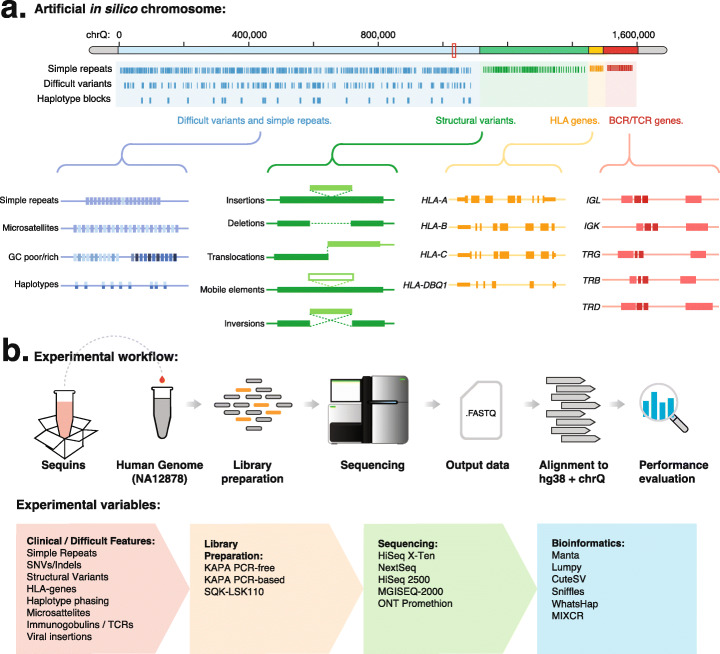
In silico chromosome design and experimental workflow. **a** The in silico decoy chromosome is designed to incorporate difficult and clinically important features of the human genome. The chromosome is divided into (i) small variants (including SNPs and indels) and simple repeats, (ii) structural variants (including large insertions, deletions, duplications, inversions, and translocations), (iii) HLA genes, and (iv) immune receptor genes. **b** The schematic diagram illustrates the use of synthetic DNA controls (sequins) and the in silico chromosome during the NGS workflow (upper panel). The range of experimental variables evaluated within this study, including difficult genetic features, library preparation methods, sequencing instruments, and bioinformatic tools, are indicated (lower panel)

Here, we designed sequins to represent difficult regions of the human genome. We first designed an in silico decoy chromosome that encoded a representative selection of difficult and clinically important regions of the human genome (Fig. 1). Regions were selected to include a wide representation of difficult sequences (such as different repeat lengths and sequence complexities) or to represent important sequences currently used in clinical diagnosis (such as microsatellites in the Bethesda panel) [17]. These difficult chromosomal sequences were then synthesized into DNA fragments that could be added to reference DNA samples and sequenced using a range of different technologies. By adding sequins to reference samples, they accumulate the same errors and bias as they proceed through the NGS workflow and can be analyzed in the output library files as internal controls. Therefore, sequins provide a ground-truth evaluation of difficult-to-sequence yet clinically important regions represented within an in silico decoy chromosome.

Using this approach, we evaluated a range of different short- and long-read sequencing technologies, library preparation methods, and software tools. We benchmarked the performance of each genome technology and performed an analytical validation for the diagnosis of clinical features in difficult regions of the genome. This provides insight into the relative strengths and weaknesses of each approach and informs the use of NGS in clinical diagnostics. We provide these sequins as a reference material for use by the research and clinical genomics community (www.sequinstandards.com) and present our evaluation of current genome technologies to inform ongoing improvements to diagnostic accuracy and yield in the remaining difficult regions of the human genome.

## Results

### Assembly of an in silico decoy chromosome

We assembled an in silico chromosome that encodes difficult and clinically important regions of the human genome represented by sequins. This artificial decoy chromosome sequence (termed *chrQ*) is designed to accompany the reference human genome (such as *hg38*) during indexing and alignment and encodes the genetic features represented by sequins in a single contiguous sequence. The in silico chromosome is approximately 1.7 Mb in length and is organized into four main functional parts, including small variants, structural variants, HLA genes, and T and B cell immune receptor genes (Fig. 1a; see Additional files 1 and 2).

The first section of the chromosome encodes a range of synthetic variants, including SNPs and indels (*n* = 1353) associated with repetitive sequences and GC-rich/poor regions, and clinically relevant microsatellites (*n* = 12). In addition, the sequins representing germline variation are produced in pairs (reference and variant; *n* = 24 pairs) to emulate the diploid alleles of the human genome. This enables the evaluation of phasing methods to correctly resolve broad haplotype blocks sampled from each human chromosome (chr1-22, X and Y; average length = 5.9Kb). The second section encodes a range of large structural variants, including deletions, insertions, duplications, inversions (including mobile element insertions), and translocations (*n* = 45). The third section encodes a range of alternative HLA alleles (*n* = 8), while the final fourth section encodes synthetic T and B cell receptor loci that have undergone V(D)J recombination (*n* = 20). Together, the in silico chromosome serves as a ground-truth reference sequence that encodes a wide range of difficult and clinically important features selected from the human genome (see Additional file 3: Table S1).

Genetic reference and variant standards represented within the in silico chromosome were first synthesized as DNA fragments (average length = 2 kb) by a commercial vendor and validated using Sanger sequencing (see Additional file 4). These sequins were then mixed at different concentrations to emulate different allele frequencies, including germline homozygous and heterozygous genotypes, but also somatic allele frequencies (Fig. 1b; see Additional file 5). This final mixture was then sequenced alone or added at a fractional concentration (~ 2%) alongside the reference human genomic DNA using a range of library preparation or sequencing technologies (NA12878; Fig. 1b).

### Study evaluation of genome technologies

Sequins provide a universal reference material to benchmark the performance of different genome technologies. We identified key variables that are known to impact performance in sequencing experiments, including base-calling accuracy, read length, or the use of PCR amplification during library preparation. We then designed experiments based on alternative preparation methods and sequencing instruments to include these key variables and evaluate the use of the in silico chromosome in diverse experimental settings. For example, we selected library preparation methods that differ in their fragmentation strategy and use of PCR amplification which can add further errors and bias during library preparation (LSK110 kit, *KAPA HyperPlus* PCR-based/PCR-free kits, and MGIEasy PCR-free; see the “Methods” section; Fig. 1a). We also considered different sequencing instruments that vary in terms of cost, read length, error rate, and throughput, such as short-read (including *Illumina HiSeq 2500™*, *HiSeq X Ten™*, *NextSeq™*, and *BGI MGISEQ-2000™*) and long-read (*Oxford Nanopore Technologies PromethION™*) sequencing technologies (Fig. 1b). Furthermore, we also prepared the standards mixture neat, without any accompanying genomic DNA, with the same preparation kit (*KAPA HyperPlus* PCR-based), but sequenced in different instruments (*HiSeq 2500* and *NextSeq*) to evaluate any instrument-specific biases (2 replicates each). Following sequencing, reads were aligned to the combined reference genome (comprising both hg38 and chrQ, see the “Methods” section), and we then employed a range of different bioinformatic tools to evaluate the alternative analytical strategies that are used to resolve difficult regions of the in silico chromosome (Fig. 1a; see Additional file 6: Table S2).

### Comparison between NA12878 genome and corresponding sequins

To initially validate the sequins, we first compared their sequencing performance to high-confidence regions and variants within the accompanying NA12878 genome sample. We showed that alignment coverage and distribution match closely between NA12878 and the accompanying standards (RMSE; *Illumina* = 0.24, *MGI* = 0.30, *ONT* = 0.18; see Fig. S1a). We next found that the sequencing mismatch error was also similar between sequins and corresponding human genome regions (RMSE; *Illumina* = 0.47, *MGI* = 0.48, *ONT* = 0.24; see Fig. S1b). The standards also reproduced errors and biases observed at more complex variants, such as large deletions that have been characterized with high confidence for NA12878 (see Fig. S2a). The commutability between sequins with NA12878 supports their use in characterizing sequencing performance in low-confidence regions and complex variants.

While the NA1878 genome and sequins exhibited similar performance in high-confidence regions and for simple variants, we found that the error profiles were different at genomic positions where NA12878 diverged from the reference genome. For example, within *Illumina HiSeq* libraries, the error frequencies at those divergent positions were higher in NA12878 alignments than corresponding sequins (single base mismatches: 3.1% in NA12878, 0.6% in sequins; insertions: 10.8% in NA12878, 0.3% in sequins; and deletions: 17.1% in NA12878, 0.3% in sequins). While sequins provide an unambiguous measurement of error at difficult sites, such as microsatellites or simple repeats, the measurement of error using the NA12878 genome is confounded by the presence of bonafide variants that cannot be reliably distinguished from sequencing errors (see Fig. S2b). This illustrates the value of sequins in providing an unambiguous representation of difficult regions. Accordingly, within the following sections, we use sequins to provide a detailed understanding of sequencing performance for these difficult regions and complex variants.

### Sequencing errors at difficult or repetitive chromosomal regions

**Fig. 2:**
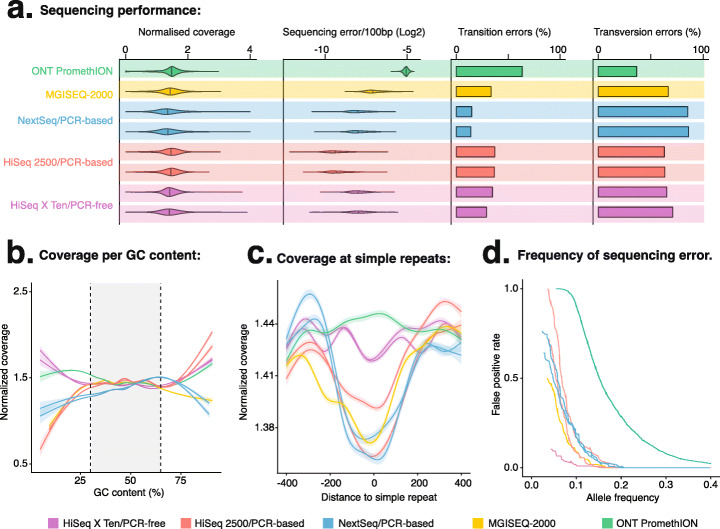
Sequencing performance metrics using sequins. **a** (left panel) Per-base normalized coverage and absolute sequencing error distributions for different sequencing libraries. (central and right panel) The relative frequency of transitions and transversions mismatch errors. **b** Average normalized coverage of libraries relative to GC content. **c** Average normalized coverage of libraries relative to simple repeats. **d** Erroneous false-positive rate of variant detection (SNVs and indels) relative to allele frequency. **a**–**d** Colors represent the different sequencing technologies/preparation methods to generate each of the seven sequenced libraries, such as *HiSeq X Ten*/PCR-free (purple), *HiSeq 2500*/PCR-based (red), *NextSeq 500*/PCR-based (blue), *MGISEQ-2000* (yellow), and *ONT PromethION* (green)

The depth and uniformity of alignment fold coverage are key variables in the detection of genetic variants. To first compare the alignment coverage of each library, we measured per-base normalized coverage across the in silico chromosome (Fig. 2a). We found that PCR-free library preparation (*IQR* = 0.35) and long-read sequencing (*IQR* = 0.30) strategies achieved the most homogenous coverage, as apparent by their lower interquartile range (IQR), while short-read PCR-based exhibited a more heterogenous coverage (*IQR*; *MGISEQ-2000* = 0.38, *HiSeq 2500* = 0.36, *NextSeq 500* = 0.46; Fig. 2a).

To identify the source of this variability in alignment coverage, we undertook a closer analysis of alignments at difficult regions of low (< 30%) or high (> 65%) GC content (Fig. 2b; see Fig. S1c). PCR-free library preparation and long-read sequencing, which achieved a globally homogenous coverage, were little impacted by GC-rich/poor regions. However, among the other technologies, there was a reduction in coverage at low GC regions for *MGISEQ-2000* (43.84% relative to mean global coverage) and *HiSeq 2500* (51.49%) (Fig. 2b; see Fig. S1c). At high GC regions, the PCR-free and *HiSeq 2500* libraries exhibited an increase in coverage with GC content (31.96% and 52.67%, respectively), while the *MGISEQ-2000* and *NextSeq 500* libraries exhibited a reduction in coverage as the GC content increased (32.08% and 28.14%, respectively; Fig. 2b; see Fig. S1c). These same libraries also showed a reduction in alignment coverage at simple repeats; however, this was less pronounced than at GC-rich/poor regions (Fig. 2c).

We next used the in silico chromosome as a ground-truth reference to measure the sequencing errors of each technology. As expected, the *ONT* long-read sequencing suffered from substantially higher error than other technologies (0.030 mismatches/kb), and among short-read instruments, the *HiSeq 2500* achieved the most accurate reads (0.0018 mismatches/kb), compared to *MGISEQ-2000* (0.0084 mismatches/kb; Fig. 2a). The relative frequency of transition and transversion errors also varied between instruments. For example, transition errors were higher for *ONT* (63.4%) and lower for *NextSeq 500* (14.4%) compared to other libraries (overall mean; transitions = 32.9% ± 15.5; Fig. 2a). Accordingly, we generated detailed sequencing error profiles for different technologies that can provide a background against which to correct mutational signatures, especially for low-frequency somatic variants (see Fig. S1d).

We next considered the impact of sequencing errors and coverage on the detection of somatic variants. For each library, we evaluated the frequency of erroneous false-positive variants that otherwise impose a lower limit on the accurate detection of low-frequency mutations. Among short-read libraries, PCR-free library preparation achieved significantly lower false discovery rates (*AUC* = 0.0035) than corresponding PCR-based preparations (*AUC*; *HiSeq 2500* = 0.035, *NextSeq 500* = 0.039) or *MGISEQ-2000* (*AUC* = 0.02). In contrast, the lower sequencing accuracy of long-read sequencing results in higher false discovery rates for somatic variants (*AUC* = 0.12). These results indicate how variation in key variables, such as coverage and sequencing error, at difficult genomic regions by different library preparation or sequencing instruments can limit the detection of clinically important features such as somatic mutations (Fig. 2d).

### Resolution of genetic variation at low-complexity regions, including microsatellites

**Fig. 3:**
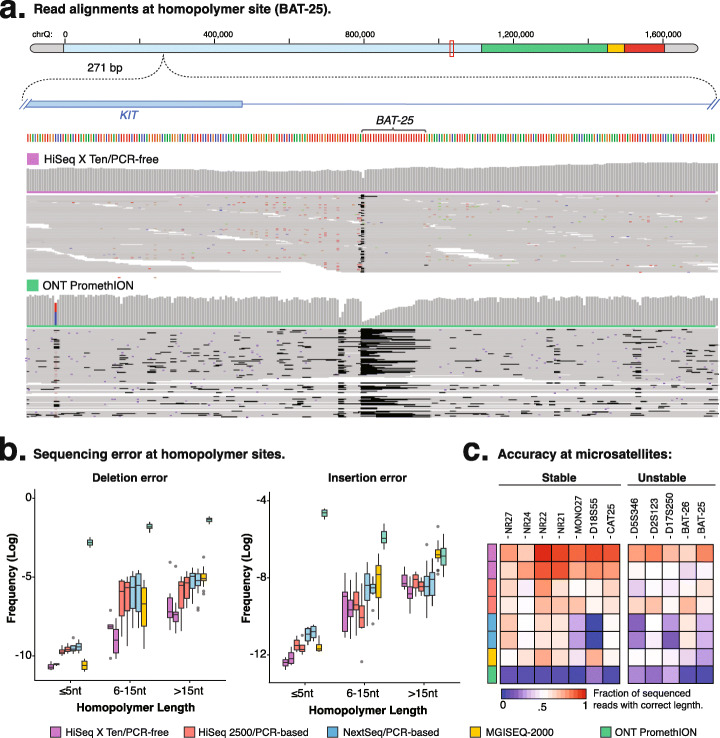
Sequencing performance at microsatellites. **a** Read alignment, for *HiSeq X Ten/PCR-free* and *ONT PromethION*, at the synthetic unstable homopolymer *BAT-25* microsatellite. The histogram shows the depth of coverage at each position, with aligned reads shown below. Any deletions in the aligned reads are indicated by black segments. **b** Frequency (log_2_) of deletions and insertions at small (≤ 5 nt), medium (> 5 nt and ≤ 15 nt), and large (> 15 nt) homopolymers. **c** Relative frequency of reads containing exact repeat size matches for the microsatellites in the Bethesda panel. **a**–**c** The colors represent the different sequencing technologies/preparation methods used to generate each of the seven sequenced libraries, such as *HiSeq X Ten*/PCR-free (purple), *HiSeq 2500*/PCR-based (red), *NextSeq 500*/PCR-based (blue), *MGISEQ-2000* (yellow), and *ONT PromethION* (green)

DNA replication of simple repeat sequences is difficult, resulting in the accumulation of mutations at these sites which, as a result, are highly polymorphic in human populations [18–20]. These simple repeats are also a challenge to sequence accurately as these technical sequencing errors can be difficult to distinguish from the biological genetic variants (Fig. 3a). Therefore, we next evaluated the detection of insertion and deletion errors at small (≤ 5 nt), medium (6–15 nt), and large (> 15 nt) homopolymer sites in the in silico chromosome across different genome technologies (see Fig. S3a).

To evaluate the performance of sequencing repeats, we first compared the fraction of reads with correct or erroneous repeat length within each library. We found that erroneous deletions (*HiSeq X Ten*/PCR-free = 3.14 × 10^−5^; *MGISEQ-2000* = 6.98 × 10^−5^; *ONT PromethION* = 5.5 × 10^−2^; Fig. 3b) are more common at homopolymer sites than insertions (*HiSeq X Ten*/PCR-free = 6.29 × 10^−6^; *MGISEQ-2000* = 2.13 × 10^−5^; *ONT PromethION* = 1.1 × 10^−2^; Fig. 3b). Furthermore, the frequency of both error types increases with homopolymer length up until ~ 15 nt, beyond which the error rates remain constant for larger repeats (mean Pearson’s correlation; deletions = 0.85 and insertions = 0.40; see Figs. S3b, c). However, for *ONT PromethION*, the rate of insertions decreases with homopolymer length (Pearson’s correlation = −0.97; see Fig. S3c). Notably, we also observed substantial performance differences due to the library preparation method and sequencing technology (Kruskal-Wallis test; *H*(7) = 94.54, *p*-value ≤ 0.0001, *N* = 31, Fig. 3b).

The difficulty in sequencing homopolymers with *ONT PromethION* is well-established, and only a minority (~ 6%) of sequenced long reads exhibited the correct length of homopolymers (see Figs. S3d, e). In contrast, among short-read libraries, PCR-free preparation significantly reduces erroneous deletion rates across all homopolymer lengths and insertion rates at small homopolymers (Fig. 3b; see Fig. S3f). Reads from PCR-free libraries also exhibit a higher proportion of exact matches (~ 77.6%) for observed homopolymer lengths compared to the other libraries (61.7%), with *MGISEQ-2000* exhibiting comparable deletion rates to PCR-free libraries at small homopolymers (Fig. 3b; see Figs. S3e, f).

Microsatellites are highly polymorphic short repeat sequences that are interspersed throughout the human genome, and are commonly used as markers in forensics and genealogy, as well as for the detection of deficient DNA mismatch repair in human diseases [21]. We designed 7 stable synthetic microsatellites (*NR27*, *NR24*, *NR22*, *NR21*, *MONO27*, *D18S55*, and *CAT25*) and 5 unstable (*BAT-25* and *BAT-26* and three dinucleotide loci *D2S123*, *D5S346*, and *D17S250*) microsatellites from the Bethesda panel (Fig. 3a) [17]. At stable microsatellites, reads should exactly match the expected microsatellite length, while reads at unstable microsatellites should vary from the expected microsatellite length (Fig. 3a; see Fig. S3g). Again, PCR-free preparation achieved the best accuracy for most stable microsatellites (82.0%); however, performance varied across the instruments (*HiSeq 2500* = 60.0%, *NextSeq 500* = 45.0%, *MGISEQ-2000* = 59.0%), with each technology exhibiting distinct biases. Finally, *ONT* long reads were largely unable to accurately resolve almost any microsatellites (6%; Fig. 3c).

In summary, we found that *ONT* is not suitable for the analysis of simple repeats due to high error rates, and, among short-red libraries, PCR amplification reduced accuracy substantially. The use of rolling circle amplification (within *MGISEQ-2000* preparation), which employs the original copy of the DNA as a template during each amplification round, exhibits better performance at small homopolymers, but remains susceptible to insertion/deletion errors at larger repeats, such as microsatellites. In summary, the exclusion of amplification in PCR-free preparation methods achieved the best performance and is likely required for the accurate detection of microsatellite instability.

### Resolution of synthetic structural variants with next-generation sequencing

Structural variants (SVs) involve the rearrangement of large chromosomal regions and can be difficult to resolve using next-generation sequencing, and the annotation of current genome references being largely restricted to insertions and deletions in high-confidence regions of the human genome [22]. Therefore, we designed a set of sequins that represented insertions (*n* = 6) and deletions (*n* = 10), but also inversions (*n* = 10), duplications (*n* = 11), viral insertions (*n* = 9), and reciprocal translocations (*n* = 8) that can benchmark the precision of structural variant detection (see Additional file 8: Table S3). To evaluate the detection of synthetic SVs, we used different software for short-read (*Lumpy* [23] and *Manta* [24]) and long-read libraries (*CuteSV* [25] and *Sniffles* [1]). The performance was evaluated according to the correct identification of the SV and the accuracy of breakpoint detection.

We first measured the performance across library preparation/sequencing technologies by aggregating the results from different structural variant callers (see Fig. S4a). We found the depth of coverage impacted sensitivity, with short-read libraries achieving better performance compared to long-read libraries when considering all the different SV types (see Fig. S4b). Similarly, among PCR-based libraries, we observe a difference between instruments, with *HiSeq 2500* performing better than *NextSeq 500* at higher coverage (two-sample Wilcoxon test; *p*-value ≤ 0.01; see Fig. S4a), while both performed equally poorly at lower coverage (see Fig. S4b).

We next evaluated the breakpoint detection achieved by the different SV software tools. For long-read *ONT* sequencing, which is able to align across large variants, *CuteSV* and *Sniffles* achieved similar overall precision (AUC; *CuteSV* = 0.64, *Sniffles* = 0.68; see Fig. S4e); however, *Sniffles* had better overall sensitivity (*AUC*; CuteSV = 0.31, Sniffles = 0.39; see Fig. S4d). The precision of breakpoint detection was high across all library preparation/sequencing technologies, with an average of 97.92% for short-read libraries, while *ONT* long-read sequencing correctly detected most breakpoints (86.77%) within 5 nt of the original position (see Fig. S4c).

We also used the synthetic structural variants to evaluate popular bioinformatic tools that identify SVs from short-read libraries. We assessed the sensitivity of these tools at varying alignment fold coverage, finding *Lumpy* and *Manta* achieved similar sensitivities (relative to fold coverage) across the libraries (*AUC*; *Lumpy* = 0.52, *Manta* = 0.51); however, *Manta* exhibited greater precision (*AUC*; *Lumpy* = 0.84, *Manta* = 0.93). Both software use split-read and discordant read-pair evidence; however, while *Lumpy* also includes read-depth into a probabilistic framework [23], *Manta* first assembles a graph of all break-end associations [24]. A direct comparison of the supporting evidence for individual SV calls showed *Manta* recovered a greater number of split-reads and discordant read-pairs that may account for the higher observed precision (see Figs. S5a, b**)**.

**Fig. 4:**
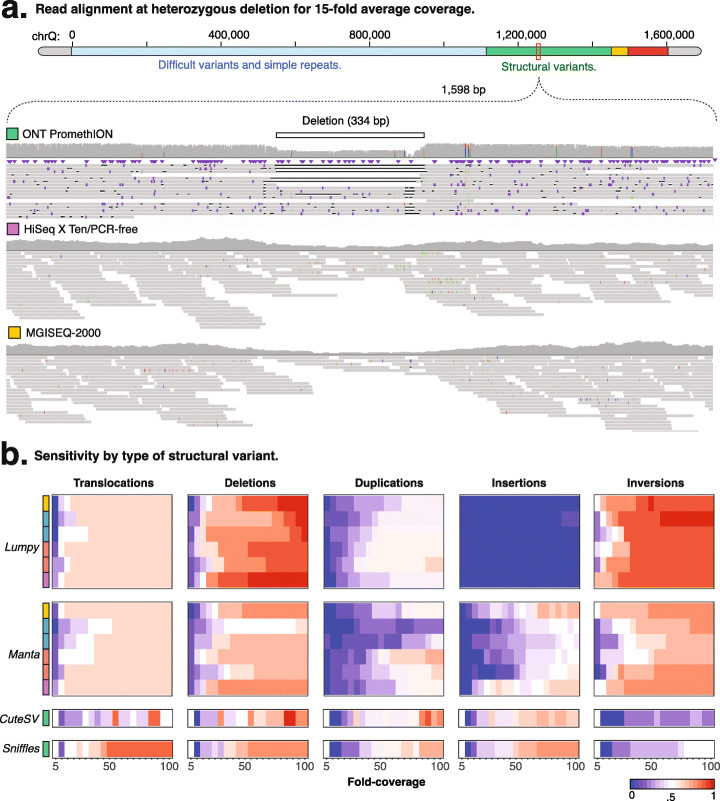
Structural variant calling performance. **a** Genome-browser view shows read alignments at deletion for *ONT PromethION* (green track), *HiSeq X Ten*/PCR-free (purple track), and *MGISEQ-2000* (yellow track) at heterozygous deletion (in white). The histogram shows the depth of coverage at each position, with aligned reads shown below. **b** Relative frequency of correctly called SVs, with breakpoints successfully identified, relative to sequencing coverage and bioinformatic tool. **a**, **b** The colors represent the different sequencing technologies/preparation methods used to generate each of the seven sequenced libraries, such as *HiSeq X Ten*/PCR-free (purple), *HiSeq 2500*/PCR-based (red), *NextSeq 500*/PCR-based (blue), *MGISEQ-2000* (yellow), and *ONT PromethION* (green)

We next investigated the ability to detect different structural variant types. Deletions, inversions, and reciprocal translocations were widely detected by the different libraries and bioinformatic tools (mean sensitivity; *DEL* = 0.67 ± 0.24, *INV* = 0.67 ± 0.26, *TRA* = 0.57 ± 0.16; Fig. 4b; see Fig. S5c). Notably, deletions and inversions had better detection among short-read libraries, while *ONT* long reads achieved better sensitivity at detecting translocations (Fig. 4a; see Fig. S5d**)**. In contrast, duplications and insertions were more challenging to detect, with long reads performing slightly better especially as the depth of coverage decreased (mean sensitivity; *DUP* = 0.35 ± 0.21, *INS* = 0.22 ± 0.26; see Fig. S5c). Overall, insertions performed poorly among PCR-based methods, while duplications had a low sensitivity particularly with *NextSeq 500* libraries (Fig. 4b). Notably, the performance also varied according to SV length, with *ONT* long-read sequencing failing to detect longer insertions that were otherwise detected within short-read libraries (see Figs. S5e, f).

Together, these results highlight the difficulty associated with SV calling, and the pervasive impact of library preparation, sequencing technology, and software on analysis. Among short-read libraries, PCR-based methods impair SV detection, while long-read sequencing can provide alignments that span large chromosomal rearrangements, and thereby resolve complex structural variants. However, all methods exhibit variable performance across the diversity of SV types. As a result, no single approach achieved comprehensive SV detection, and instead, a combination of approaches was required to identify the range of ground-truth synthetic SVs.

### Phasing genetic variants into haplotype blocks

The phasing of alleles enables genetic variants to be linked to paternal or maternal human chromosomes [26]. However, phasing can be difficult at regions with sparse variants and be limited by fragment size and read length. To evaluate the phasing accuracy achieved by different library preparation and sequencing technologies, we designed 22 pairs of sequins that each represent maternal and paternal alleles for large regions (~ 6 kb) of each human chromosome, as well as chromosomes X and Y. Each control pair includes allele-specific common genetic variation and forms a diploid representation of human chromosomes (see Fig. S6a). To phase these synthetic alleles within NGS libraries, we used *WhatsHap* [27] and evaluated performance according to the fraction of correctly phased variants, and the length of correctly resolved haplotype blocks.

**Fig. 5:**
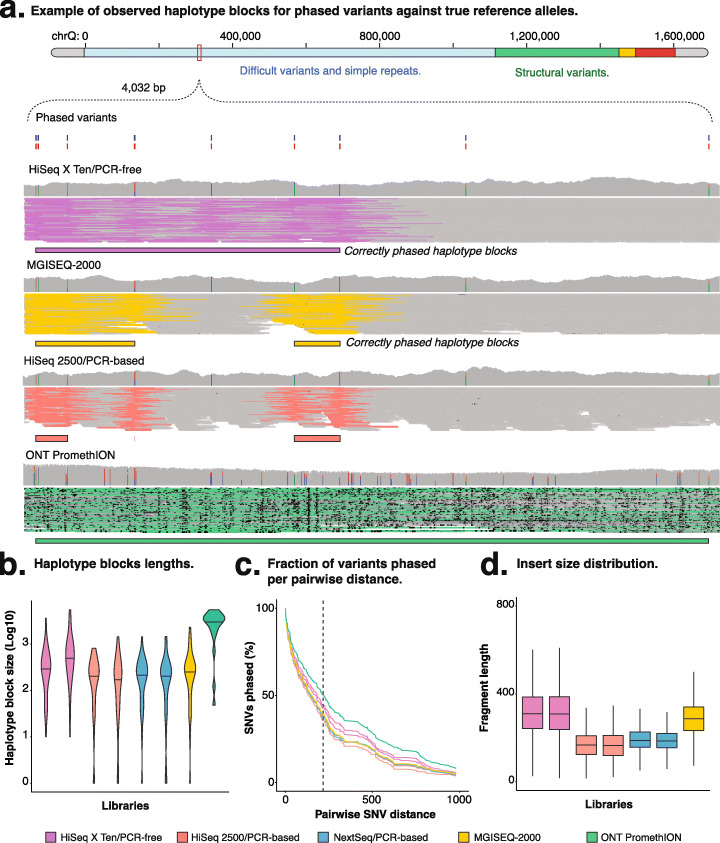
Phasing performance. **a** Read alignment at synthetic haplotype blocks with multiple heterozygous variants representing a pair of alleles from chromosome 20. The histogram shows the depth of coverage at each position, with aligned reads shown below. The assembled haplotype blocks (boxes) obtained with the different sequencing technologies/preparation methods are shown below the sequencing reads, with the reads supporting the assemblies being highlighted. **b** Distribution of observed lengths for resolved haplotype blocks according to different sequencing technologies/preparation methods. **c** Fraction of phased variants relative to pairwise variant distance. **d** Size distribution for DNA fragments in the short-read libraries. **a**, **b** The colors represent the different sequencing technologies/preparation methods used to generate each of the seven sequenced libraries, such as *HiSeq X Ten*/PCR-free (purple), *HiSeq 2500*/PCR-based (red), *NextSeq 500*/PCR-based (blue), *MGISEQ-2000* (yellow), and *ONT PromethION* (green)

The initial inspection of read alignments reveals clear differences in phased haplotype blocks between short- and long-read libraries. For example, phasing synthetic heterozygous variants on chromosome 20 revealed progressively longer haplotype blocks for *ONT*, *HiSeq X Ten/*PCR-free, *MGISEQ-2000*, and *HiSeq 2500/*PCR-based (Fig. 5a). Indeed, *ONT* achieved significantly longer blocks (see Fig. S6b) overall compared to all other technologies. The average read length for *ONT* was 755.3 nt (*SD* = 831.6; see Fig. S6c), which was limited by the length of sequins (~ 2 kb on average; see the “Methods” section), and long-read technology was capable of consistently phasing distant variants (> 1000 nt apart; see Fig. S6d) that cannot be otherwise phased with short-read libraries (Fig. 5c). These longer haplotypes generated by long-read *ONT* sequencing exhibited slightly lower sensitivity (long-read = 0.93, short-read = 0.98 ± 0.01), but also a lower proportion of false-positive variants compared to short-read PCR-based methods (long-read = 3.03%, average short-read/PCR-based = 7.67%; see Fig. S6e).

Within short-read libraries, PCR-free preparation achieved longer haplotype blocks than alternative PCR-based methods (pairwise Mann-Whitney-Wilcoxon test; Mann-Whitney-Wilcoxon test; *p*-value ≤ 0.001; Fig. 5b; see Fig. S6b). Given that phasing accuracy is a function of the pairwise distance between variants, we found that this advantage was most apparent in non-polymorphic genome regions, where variants are sparsely distributed (Fig. 5c; see Figs. S6f, g). This advantage was supported by the distribution of DNA insert size which showed that PCR-based libraries had smaller DNA fragments (*HiSeq 2500* = 168.83, *NextSeq 500* = 195.16) than PCR-free or *MGISEQ-2000* libraries (*HiSeq X Ten* = 319.82, *MGISEQ-2000* = 289.63; Fig. 5d). Indeed, while the DNA fragment size distribution of these approaches had similar medians (*HiSeq X Ten* = 308, *MGISEQ-2000* = 287), there was a subset of longer fragments (19.79%) in the PCR-free library which enabled phasing of more distant variants (Fig. 5a, d**)**.

Together, this analysis illustrates the importance of longer DNA fragment size and read length, as well as variant density, required to achieve successful phasing. Furthermore, the gap between shorter haplotypes with more accurate variant detection provided by short-read sequencing and less accurate but longer haplotypes provided by long-read *ONT* sequencing continues to close. Optimally, a combination of these two sequencing technologies should produce longer, but more accurate phased haplotypes.

### Impact of sequencing accuracy and coverage on HLA typing

The recognition of non-self-antigens by the immune system is mediated through the major histocompatibility complex (MHC) which is encoded within a 3.6-Mb region on chromosome 6. Due to selective pressures, this is one of the most polymorphic loci in the human genome, and variation of the human leukocyte antigen (HLA) genes is associated with disease [28, 29]. The accurate and rapid resolution of HLA genes is also required for successful donor-patient matching in organ transplantation. However, due to the complexity and hypervariability of this region, the accurate typing of HLA genes remains difficult with NGS [30].

**Fig. 6:**
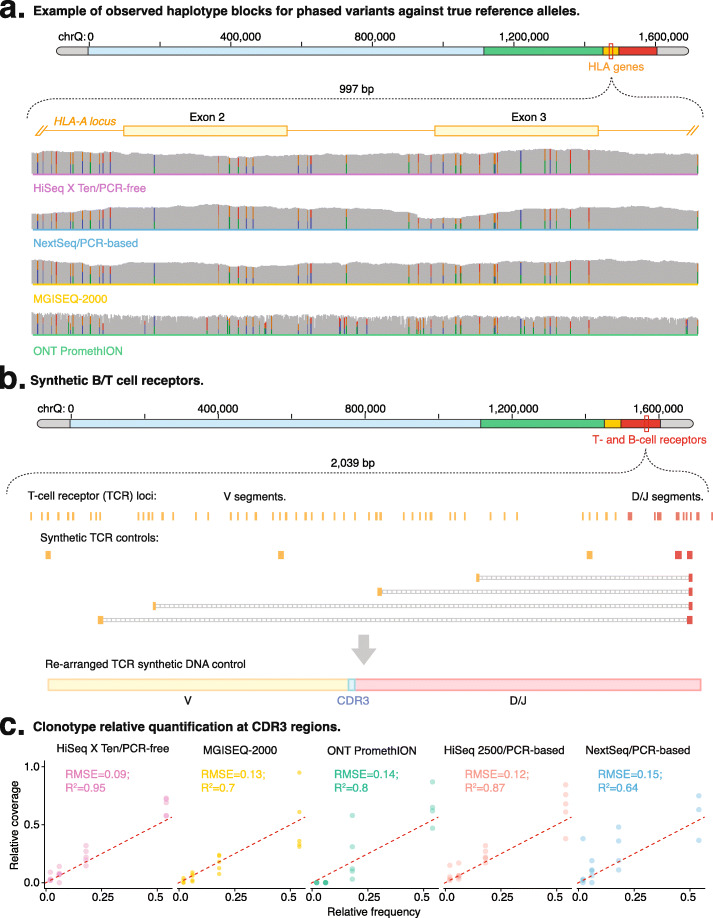
HLA typing performance. **a** Read alignment for *Hiseq X Ten*/PCR-free (purple), *Nextseq 500*/PCR-based (blue), MGISEQ-2000 (yellow), and *ONT PromethION* (green) at exons 2 and 3 (G-group) of the HLA-A gene representation of the in silico chromosome. Immune repertoire analysis. **b** The schematic figure indicates the design of sequins for the *TCRB* gene loci. **c** Observed versus expected clonotype frequencies for quantification of the *CDR3* region at different TCRs and BCRs, with dashed lines indicating a slope of 1

To evaluate the use of NGS to perform accurate HLA typing, we incorporated a synthetic *MHC* region within the in silico chromosome that was accompanied by sequins representing *HLA-A*, *HLA-B*, *HLA-C*, and *HLA-DQB1* alleles (Fig. 6a). We first inspected alignment accuracy at the reference *HLA* genes on the in silico chromosome (Fig. 6a). We found that short-read libraries closely matched the sequins, while *ONT* long-read sequencing, which exhibits an elevated sequencing error rate (mean 5-fold; see Fig. S7a), performed comparably to other technologies at the consensus level, with no errors observed within exons 2 and 3 of *HLA-C* and *HLA-B* (see Fig. S7b).

We then focused on typing the G-group exons (2 and 3) using *HLA-LA* [31] at varying fold coverage. At the antigen level (where different alleles expressing the same epitopes are grouped together), all libraries achieved accurate typing of the *HLA* alleles (see Figs. S7c, d). However, at the allele-group resolution level, which is a more specific standard to evaluate HLA typing, we observed variation in library performance that was largely dependent on coverage depth (see Fig. S7e). At 25-fold coverage or higher, HLA-typing accuracy is comparable among all short-read libraries, while PCR-free and *NextSeq 500* libraries achieved the best sensitivity at lower coverage (down to 5-fold) due to increased coverage at the target exons used for HLA typing (see Figs. S7c, e).

*ONT* sequencing provides rapid, real-time sequencing that can rapidly match donor transplants to a recipient host during surgery and has accordingly been of considerable interest for HLA typing [32]. We found that at high-depth coverage, *ONT* genotyping performs comparably with other short-read sequencing approaches. However, at coverages lower than 25-fold, we observed that *ONT* consistently misdiagnosed allele groups, and the lower base-calling accuracy of *ONT* confounds accurate determination of genotypes (Fig. S7c).

### Clonotype repertoire analysis of synthetic immune receptors

T cell receptors (TCR) and B cell receptors/immunoglobulins (BCR/Ig) recognize diverse foreign antigens as part of an effective adaptive immune response. These TCR and BCR genes undergo stochastic V(D)J recombination to generate a massive combinatorial diversity of receptor sequences that is further increased by random nucleotide excision and addition at rearranged junctions [33]. NGS is being increasingly used to profile this repertoire of TCR and BCR clonotypes and measure T- and B cell dynamics in healthy individuals, patients with cancer, infections, and autoimmune diseases. However, these diverse applications have different requirements for sensitivity, specificity, and quantitative accuracy that are impacted by sequencing and analytical errors [34, 35].

To evaluate the genome DNA-based profiling of TCR and BCR genes using NGS, we developed a synthetic immune repertoire containing both non-rearranged and rearranged *IGL*, *IGK*, *TRG*, *TRD*, and *TRB* clonotypes (Fig. 6b). These synthetic clonotypes represent TCR and BCR sequences that were derived from patient samples [36]. To further evaluate the quantitative accuracy of profiling techniques, we also mixed the sequins at different concentrations to form a quantitative ladder that encompasses different clonotype frequencies.

We first measured the sequencing accuracy of *CDR3* sequences from rearranged TCRs and BCRs standards. Among short reads, the *HiSeq 2500* (0.28 errors/100 nt) and *NextSeq 500* (0.41 errors/100 nt) accumulated less errors than other instruments at CDR3 regions (*HiSeq X Ten* = 0.59 errors/100 nt, *MGISEQ-2000* = 0.98 errors/100 nt; see Fig. S8a), while, as expected, the greater error rate for *ONT* long-read sequencing (9.06 errors/100 nt) was insufficient for the unambiguous resolution of CDR3 sequences.

We found that the PCR-free method demonstrated the best quantification of clonotype abundances (*RMSE* = 0.09; *R*^2^ = 0.95), while *NextSeq 500* (*RMSE* = 0.15; *R*^2^ = 0.64) and *ONT* (*RMSE* = 0.14; *R*^2^ = 0.8) measured the clonotype frequencies poorly, especially at higher fractions (Fig. 6c). Notably, the ability to accurately resolve CDR3 sequences was directly correlated with clonotype frequency, with low-frequency clonotypes proportionately accumulating more errors (see Fig. S8a).

We next evaluated the accuracy by which rearranged TCR and BCR clonotypes were resolved using *MiXCR* [37]. Among short-read libraries, we observed high sensitivity for measuring clonotypes (varying between 78.9 and 89.4%), with most missed clonotypes having low expected frequency (see Fig. S8b). While CDR3 sequence was correctly identified, the recombined V(D)J segments, such as *TRBV12-4/TRBD2/TRBJ2-2*, were erroneously classified as *TRBV12-4/TRBD1/TRBJ2-2* across all short-read libraries, indicating the presence of systemic false-positive artifacts. Nevertheless, despite these errors, we generally observed high precision (varying between 85.0 and 94.4%), with most false-positive segments resulting from clonotypes that were identified at low frequency (mean false-positive frequency of 0.0038; see Fig. S8c).

While the sequins do not encompass the scope and complexity of natural immune repertoires, they do provide a ground-truth standardized reference that can identify systematic biases, even when shared by all technologies. This construction of a synthetic repertoire provides a useful reference for the standardization of immune repertoire profiling by the research community [38, 39].

## Discussion

The human genome sequence contains polymorphic, repetitive, and complex regions that are difficult to sequence and are typically ignored during analysis. However, these regions include clinically important features, and their accurate resolution can improve the diagnostic yield of clinical NGS. Here, we built sequins accompanied by an in silico decoy chromosome that incorporates these challenging sequences and provides an unambiguous reference against which to evaluate the use of NGS to diagnose difficult and clinically important human genome sequences.

The in silico chromosome is designed to accompany the human genome reference files during the alignment and analysis of DNA samples. This in silico chromosome encodes a range of genetic variants, including small SNPs and indels, as well as larger structural variants, such as duplications, inversions, and translocations. These variants can be further organized into broader haplotype blocks that emulate the paternal and maternal chromosomes of the diploid human genome. In addition to representing common genetic variation, the in silico chromosome also encodes multiple features of interest, including HLA genes and immune receptors, as well as instances of viral (HPV) insertions. We anticipate that additional genetic features can also be added to future in silico chromosome builds.

Within this study, we employed sequins to evaluate the performance of different library preparation methods, sequencing technologies, and bioinformatic software to resolve these difficult human genome regions. We provided summary statistics, such as error rate, for sequence-specific contexts for individual experiments and also compared our sequins to a reference genome sample, such as NA12878. We showed that overall the standards and NA12878 are commutable; however, higher error rates for NA12878 at several genome positions indicate possible unannotated variants in this reference sample. We also observed substantial variation between similar instruments or library preparation methods, with the sequins forming a common reference against which to benchmark these advantages and limitations.

Using this approach, we provided a direct comparison of short-read technologies on difficult genome regions that had not previously been benchmarked. We found that the use of PCR amplification within library preparation methods resulted in the accumulation of errors that was a major factor in differentiating the performance between these technologies. This included variable amplification efficiencies that caused heterogenous coverage across high/low GC regions, and resulted in the accumulation of errors at short repeats and microsatellites. The use of DNA nanoballs and rolling circle amplification (used during library preparation for the MGISEQ-2000 instrument) performed comparably to PCR-based libraries, albeit with improved resolution of short repeats and microsatellites. However, the exclusion of PCR-based steps during library preparation achieved the most uniform coverage of difficult regions, with less insertion/deletion errors as well as more comprehensive phasing, more sensitive SV breakpoint detection, and also better quantitative accuracy in an immune repertoire analysis.

As anticipated, the long-read nanopore sequencing was superior for phasing haplotype blocks and resolving some types of large structural variants. Furthermore, the full advantages of long-read sequencing may not be fully realized in our benchmarking analysis given that synthetic controls used in this study are shorter than the typical read length achieved with long-read sequencing. Nevertheless, these benefits were often offset by lower base-calling accuracy, resulting in false-positive variants that were unsuitable to detect somatic variants or evaluate microsatellite instability, and may otherwise confound analysis in whole-genome sequencing.

Furthermore, at higher coverage depth, we found that short-read libraries could similarly resolve the exact location of breakpoints for complex structural variants. Given these complementary advantages, we recommend that low-coverage long-read sequencing combined with high-coverage short-read PCR-free sequencing currently provides the most comprehensive solution to sequencing and analyzing difficult and challenging regions of the human genome. Nevertheless, despite this recommendation, we noted that many challenges remain, and the sequencing of difficult regions can still benefit from further improvements and innovations in genome technologies.

The use of sequins within this study provided an unambiguous ground-truth reference for complex genomic regions, including pathogenic variants of interest, represented at specific allele frequencies. Sequins can address gaps in current reference controls and complement natural reference genome materials recently released, such as HG002 and HG00733. Sequins confer the ability to flexibly represent pathological and/or rare mutations that are not otherwise found in healthy reference genomes, and furthermore, a single sequin mixture contains many different mutations types, while natural reference genomes may only harbor a few clinically relevant mutations each. Therefore, sequins provide a comprehensive coverage of clinically important genetic features and can be feasibly updated to incorporate new informatic biomarkers.

## Conclusions

Given the advantages of sequins, the SEQC2 consortium accordingly provides these controls as a reference material to encourage the benchmarking of additional new genome technologies by the research and clinical genomics community. These standards can also be used as a reference to improve variant detection at challenging regions of the human genome, expanding the available annotation for reference genome samples, such as NA12878. This resource also provides an open platform for validating and comparing the performance of NGS technologies to resolve difficult regions of the human genome. With the recent advent of rapid and affordable DNA synthesis, we anticipate that the creative design of additional sequins will further improve the catalog of available reference genome standards.

## Methods

### Sequin design and organization into an in silico chromosome

Due to its 5′-3′ directionality, a DNA sequence is entirely distinct from its mirror image. Sequins represent mirror images of naturally occurring DNA present in the NA12878 reference genome sample. Therefore, in a sequenced library of NA12878 genome sample spiked with sequins, we can unequivocally partition sequin reads from NA12878 reads. This design principle is articulated in detail in Deveson et al. [16]. In order to represent genetic variation, sequin controls often exist in pairs, with overlapping sequences encoding synthetic reference and variant alleles. The pairs have the same sequence except for the site of genetic variants that they encode. To represent germline heterozygous variation, reference and variant sequin molecules are added in the final sequin mixture at the same relative concentration. In contrast, to represent somatic variation, reference and variant sequin molecules are added at differing relative concentrations. For example, to represent a somatic mutation at 1% VAF, the variant sequin allele would be added at a 1:99 ratio relative to the reference sequin allele in the sequin mixture.

The in silico chromosome (chrQ) was designed by concatenating the sequences of sequins with different functional features into four different regions: difficult variants and simple repeats, structural variants, HLA genes, and immune receptor genes. For reference/variant sequin pairs, only the reference sequence was represented in the in silico chromosome. Consequently, reads from reference/variant sequin pairs would map at the same position within the in silico chromosome. However, apart from this circumstance, there is no other overlap in the in silico chromosome between sequins representing different features. Both the sequences of individual sequins and the in silico chromosome are available as supplementary material.

### Production of DNA sequin mixtures

Each DNA sequin was synthesized by a commercial vendor (*ThermoFisher-GeneArt*) and cloned into a pMA vector. The full sequence of each sequin was verified during synthesis using Sanger sequencing by the commercial vendor, and any erroneous sequences were re-constructed before shipping. The individual plasmids containing each sequin were transformed in *E. coli*, then grown in a 50-ml culture, and later purified. The sequins were excised from the plasmids; then, the size of the final sequence was confirmed on an agarose gel. Purified sequins were quantified using the *BR dsDNA Qubit Assay* on a *Qubit 2.0 Fluorometer* (*Life Technologies*), by taking the average of 3 independent measurements, and verified on the *Agilent 2100 Bioanalyzer* with the *Agilent High Sensitivity* DNA Kit (*Agilent Technologies*). Individual sequins were then combined at specific concentrations using an *epMotion 5070* liquid handling robot. Mixture stocks were prepared as single-use aliquots and stored at − 80 °C.

### Preparation of DNA libraries and sequencing

We first sequenced neat preparations of the sequin mixture. Libraries were prepared using the *KAPA HyperPlus* PCR-based kit (*Illumina*) in accordance with the manufacturer’s instructions. Prepared libraries were quantified on a *Qubit* (*Life Technologies*) and verified on the *Agilent 2100 Bioanalyzer* with the *Agilent High Sensitivity* DNA Kit (*Agilent Technologies*). Finally, two of the libraries were sequenced on a *HiSeq 2500* (*Illumina*) and the other two were sequenced on a *NextSeq 500*, producing paired reads of 125 nt and 150 nt, respectively. The sequencing was performed at the Kinghorn Centre for Clinical Genomics, Darlinghurst, New South Wales.

We next prepared libraries by adding the sequin mixture to NA12878 genomic DNA. Although sequins only need to be added at ~ 0.05% to achieve similar coverage to the human sample, we add the sequin mixture at a higher molarity than the accompanying human DNA, so reads can be subsequently down-sampled bioinformatically to any desired coverage. We prepared two libraries with the *KAPA HyperPlus* PCR-free kit. After the quantification and quality assessment with the *Qubit* (*Invitrogen*) and *Agilent 2100 Bioanalyzer* with the *Agilent High Sensitivity* DNA Kit (*Agilent Technologies*), the libraries were sequenced on a *HiSeq X Ten* (*Illumina*) producing paired reads of 150 nt at the Kinghorn Centre for Clinical Genomics, Darlinghurst, New South Wales. Additionally, we also prepared a library for nanopore sequencing, with the LSK110 kit (1D ligation) according to the manufacturer’s instructions and sequenced on a *PromethION* instrument, at the Kinghorn Centre for Clinical Genomics, Darlinghurst, New South Wales. Base-calling was achieved using ONT Guppy Software (version 4.5.3). Finally, we sent a sample, combining the sequin mixture and NA12878, to be prepared and sequenced in a *MGISeq2000*, by *BGI Tech Solutions*, in Hong Kong.

### Alignment to reference

We initially prepared a combined reference file containing the human reference genome (hg38) and the in silico chromosome (without genetic variation). We then indexed the reference with the BWA index and aligned with the *BWA mem* algorithm. Default parameters were used in all the analyses. For samples containing both the sequin mixture and NA12878, we used *Anaquin* [40] to partition and flipped sequin reads to be in the same orientation of hg38. The sequin reads can then be re-aligned to hg38 and analyzed concomitantly to the accompanying NA12878 reads. We aligned ONT reads with minimap2 (version 2.22-r1105-dirty) with default parameters [41]. The length of sequins imposes constraints on reads mapping at the ends of the sequence. There is an “edge effect” wherein we observe a decline in sequencing coverage at sequin termini, within one sequencing fragment length of the first and last bases. To prevent this edge bias from impacting results, we exclude ~ 400 nt edge regions during analysis.

### Performance statistics

We estimated the variability for depth of coverage and sequencing error based on the small variants section of the in silico chromosome. We used *pysamstats* (version 1.1.2) to retrieve the coverage and specific error types, such as mismatches or insertions and deletions, for every genome position. The coverage was normalized so that the total read count was the same between libraries. To calculate the variation in normalized coverage relative to GC content, we established 100-bp sliding windows, and for each window, we calculated the GC content and average normalized coverage. We then compared the observed relationship between GC content and normalized coverage in each sequenced library. At each genome position, we calculated the relative frequency of mismatches, insertions, and deletions, by dividing the number of reads containing each of those errors by the total read count at that position. To amplify the signal, we calculated the average mismatch, insertion, and deletion rates at 1000-bp windows. Finally, based on the total number of mismatches observed in each library, excluding positions with germline or somatic variants, we calculated the relative frequency of every possible substitution type, also summarizing the results as the relative frequency of transitions and transversions.

### False-positive rate of detection for small variants

To perform a fair comparison across the different sequencing libraries, we used *Varscan2* (v2.4.3) to call variants [42]. In order to maximize the discovery of somatic variants at very low allele frequencies, we set filtering parameters to be very permissive. For example, we set the minimum coverage (--min-coverage) to 50, minimum number of reads (--min-reads2) to 1, minimum variant frequency (--min-var-freq) to 10^−5^, *p*-value cutoff (--*p*-value) to 10^−1^, and strand filter to 0. This strategy maximizes the variant discovery, enhancing the signal to calculate the false discovery rate for different allele frequencies.

### Microsatellites

We first identified homopolymers in the in silico chromosome, classifying them as small (≤5 nt), medium (> 5 nt and ≤15 nt), and large (> 15 nt). We then measured the rates of insertions and deletions within these homopolymer positions by using *pysamstats* (version 1.1.2). We then recovered all the reads overlapping large homopolymers or microsatellites and measured the repeat length in each read relative to the true original length present in the reference chromosome. Finally, we calculated the error distribution for microsatellite lengths and the proportional of exact matches (reads in which the observed repeat length was equal to the expected length) for microsatellites in the Bethesda panel.

### Structural variant detection

We called structural variants for the short-read libraries using *Manta* (version 1.6.0) and Lumpy (version 0.2.13) [23, 24]. We ran *Manta* with the default parameters on the BAM alignment files. *Lumpy*, however, requires split and discordant read-pairs to be provided as separate inputs. We used “*samtools view*” (version 1.9) with the option -F 1294 to extract discordant read-pairs and “*extractSplitReads_BwaMe*,” a script provided by *Lumpy*, to extract split-reads. We then used these individual subset alignment files, as well as the original alignments, to run Lumpy with default parameters. For long reads, we called structural variants using *Sniffles* (version 1.0.11) and *CuteSV* (version 1.0.12), also with default parameters [1, 25]. To evaluate the performance at the breakpoint level, we considered breakpoints identified within 10 nt from the true position to be true-positives (TP) and breakpoints identified outside of this window to be false-positives (FP). *Sniffles* and *CuteSV* call duplications as insertions, so any insertions called within the boundaries of a duplication that had the expected size were also considered true positives. Furthermore, missed calls within the 10-nt window, where true breakpoint positions existed, were considered false negatives (FN). The sensitivity at the breakpoint level was calculated as TP/(TP + FN) and the precision as TP/(TP + FP). To evaluate the performance at the SV level, we considered a true-positive, if all the individual breakpoints for a given structural variant were successfully identified; otherwise, the SV was considered a false-negative.

### Phasing

We first identified the variants present within the defined haplotype blocks. We used *GATK* (version 4.0.0.0) for the short-read libraries and *clair3* (v0.1-r7) for long reads, with default parameters [43, 44]. We then used the individual VCF files and BAM alignments as inputs to resolve haplotypes using *WhatsHap* (version 0.18), with the “phase set” (PS) tag enabled, providing unique identifiers for individual blocks [27]. We compared each of the identified haplotype blocks with the truth set to determine the proportion of heterozygous variants that were correctly phased and also the size of blocks relative to the expected length.

### HLA typing

We evaluated sequencing accuracy at HLA genes by aligning reads onto the in silico chromosome. We then used *pysamstats* (version 1.1.2) to recover read coverage and sequencing error statistics for genome positions in exons 2 and 3, which are the most commonly used for typing. We also recovered the consensus sequence for reads overlapping exons 2 and 3 and calculated the edit distance relative to the reference sequence. We also performed HLA typing agnostically to the in silico chromosome, by using the *HLA-LA* software (version 1.0.1). We used the pre-computed hg38 population reference graph provided with the software “PRG_MHC_GRCh38_withIMGT” to align the reads, and for long reads, we also used the parameter --longReads ont2d.

### Immune repertoire analysis

We first mapped reads onto the in silico chromosome. We then evaluated sequencing accuracy at the CDR3 region, defined by the conserved cysteine-104 and typtophan-118 based on the *IMGT* numbering system [36]. We recovered read coverage and sequencing error statistics at the CDR3 region by using *pysamtats* and quantified different clonotypes based on the average coverage at the CDR3 region relative to the expected frequency. Then, for short-read libraries, we independently identified clonotypes by using *MixCR* (version 3.0.12) with default parameters. We benchmarked the performance by evaluating the detected receptor, both at the level of the CDR3 region, as well as individual V(D)J segments.

### Statistical analysis

We used the Kruskal-Wallis test to determine significant differences between all evaluated datasets followed by two-tailed Mann-Whitney tests to identify significances between any pair of specific datasets. For multiple comparisons, the *p*-values were adjusted using the false discovery rate method with an alpha of 0.05. All the statistical analyses were performed in R (v3).

## Supplementary Information

**Additional file 1.** ChrQ sequence: fasta file containing the nucleotide sequence of chrQ.

**Additional file 2.** ChrQ cytoband: file containing the coordinates for the different regions of chrQ (p1) small variants (including SNPs and indels) and simple repeats, (p2) structural variants (including large insertions, deletions, duplications, inversions and translocations), (p3) HLA genes and (p4) immune receptor genes.

**Additional file 3: Table S1.** file containing the coordinates of individual sequins within chrQ.

**Additional file 4.** Sequin sequences: fasta file containing the nucleotide sequence for individual sequins.

**Additional file 5.** Sequin small variants annotation: vcf file containing the annotation of SNPs and indels present in chrQ.

**Additional file 6: Table S2.** sequencing summary statistics for sequin reads in each individual library used in this study.

**Additional file 7.** Supplementary figures: file containing supplementary figures described in the main text.

**Additional file 8: Table S3.** file providing additional information on the structural variants represented by sequins, such as the location in hg38, type and size.

**Additional file 9.** Review history.

## Data Availability

All next-generation sequencing data is available from SRA (https://www.ncbi.nlm.nih.gov/bioproject/PRJNA625156) with the PRJNA660196 accession identifier [45]*.* In silico chromosome sequences and annotations are also available from www.sequinstandards.com/resources/. *Anaquin*, the toolkit used in the sequins analyses, is freely available at https://www.sequinstandards.com, and all scripts used to perform statistical analyses and generate plots can be found at https://github.com/almreis/Benchmark_ChrQ under MIT License [46].
