# Supplementary material for: Using synthetic chromosome controls to evaluate the sequencing of difficult regions within the human genome
Source: Genome Biol. 2022 Jan 12;23:19. doi: 10.1186/s13059-021-02579-6 (PMC8753822; doi:10.1186/s13059-021-02579-6)
Supplement: Supplementary file 7 — Additional file 7. Supplementary figures: file containing supplementary figures described in the main text. [file 13059_2021_2579_MOESM7_ESM.docx]

**Using synthetic chromosome controls to evaluate the sequencing of difficult regions within the human genome**

**Andre L. M. Reis^1^, Ira W. Deveson^1,2^, Bindu Swapna Madala^3^, Ted Wong^3^, Chris Barker^3^, Joshua Xu^4^, Niall Lennon^5^, Weida Tong^4^, & Tim R. Mercer^3,6^* on behalf of the SEQC2 Consortium**

^1^ Kinghorn Centre for Clinical Genomics, Garvan Institute of Medical Research, Sydney, NSW, Australia

^2^ St Vincent’s Clinical School, University of New South Wales, Sydney, NSW, Australia

^3^ Genomics and Epigenetics Theme, Garvan Institute of Medical Research, Sydney, NSW, Australia.

^4^ Division of Bioinformatics and Biostatistics, National Center for Toxicological Research, U.S. Food and Drug Administration, Jefferson, AR 72079, United States

^5^ Broad Institute of MIT and Harvard, MA 02142, United States

^6^ Australian Institute for Biotechnology and Nanoengineering, University of Queensland, Brisbane, QLD, Australia

**
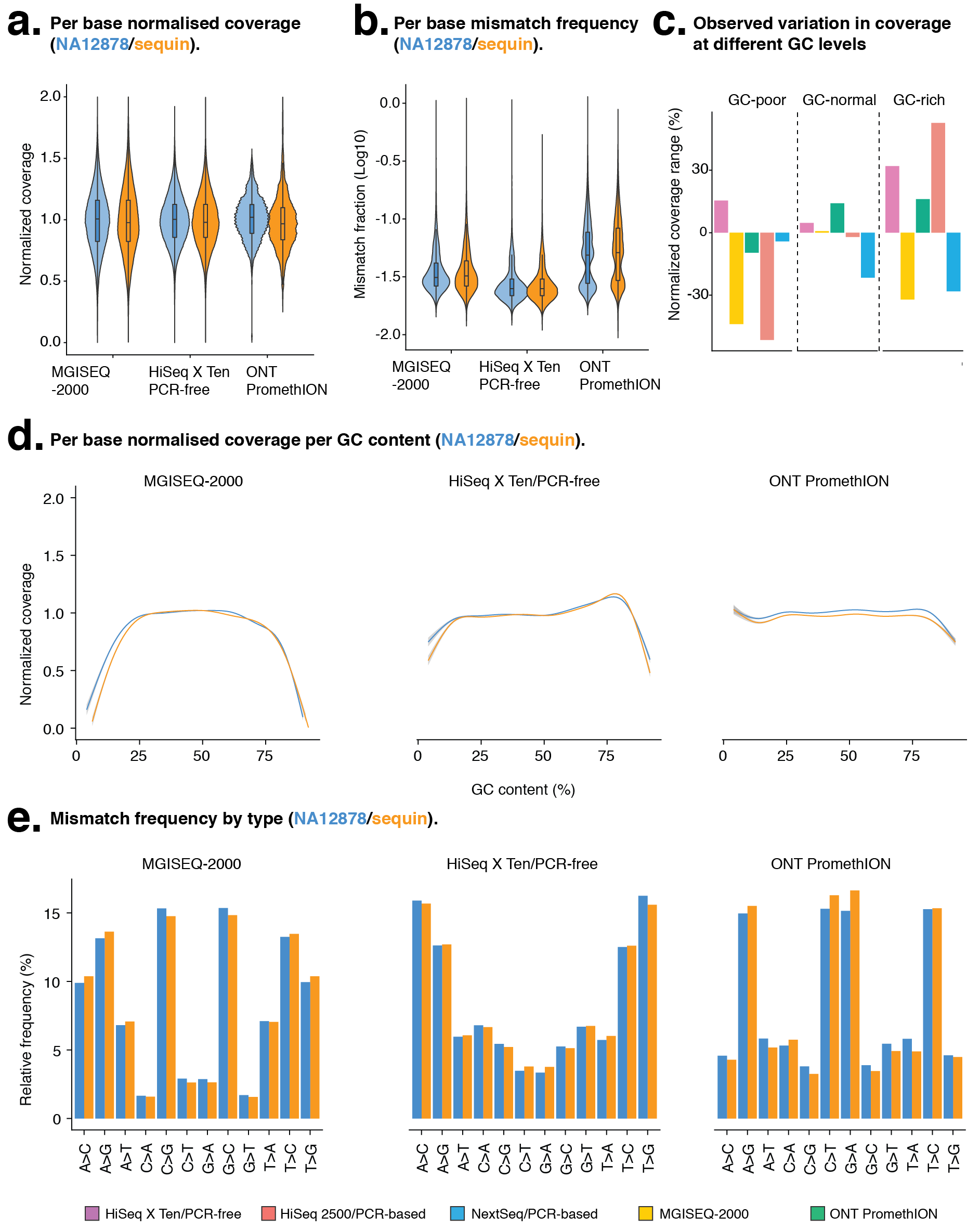
**

**Fig. S1. Commutability between NA12878 and sequins. (a-b)** Per-base normalized coverage and sequencing error distributions calculated between NA12878 high-confidence regions and their sequin counterpart for MGISEQ-200, HiSEQ X Ten/PCR-free and ONT PromethION. **(c)** Percentual variation in average normalized coverage for each technology at different GC level categories (GC-poor ≤ 0.3, GC-normal: 0.3-0.65 and GC-rich ≥ 0.65). Values smaller than 0 represent a percentual reduction in coverage and values greater than 0 represent a precentual increase in coverage. (**d)** Average normalized coverage per GC content in 100-bp sliding windows for NA12878 high-confidence regions and their sequin counterpart for MGISEQ-200, HiSEQ X Ten/PCR-free and ONT PromethION. **(e)** Relative frequency of mismatch types at NA12878 high-confidence regions and their sequin counterpart for MGISEQ-200, HiSEQ XTen/PCR-free and ONT PromethION. **(a-d)** For each library NA12878 data is represented in blue and corresponding sequin data is represented in orange.


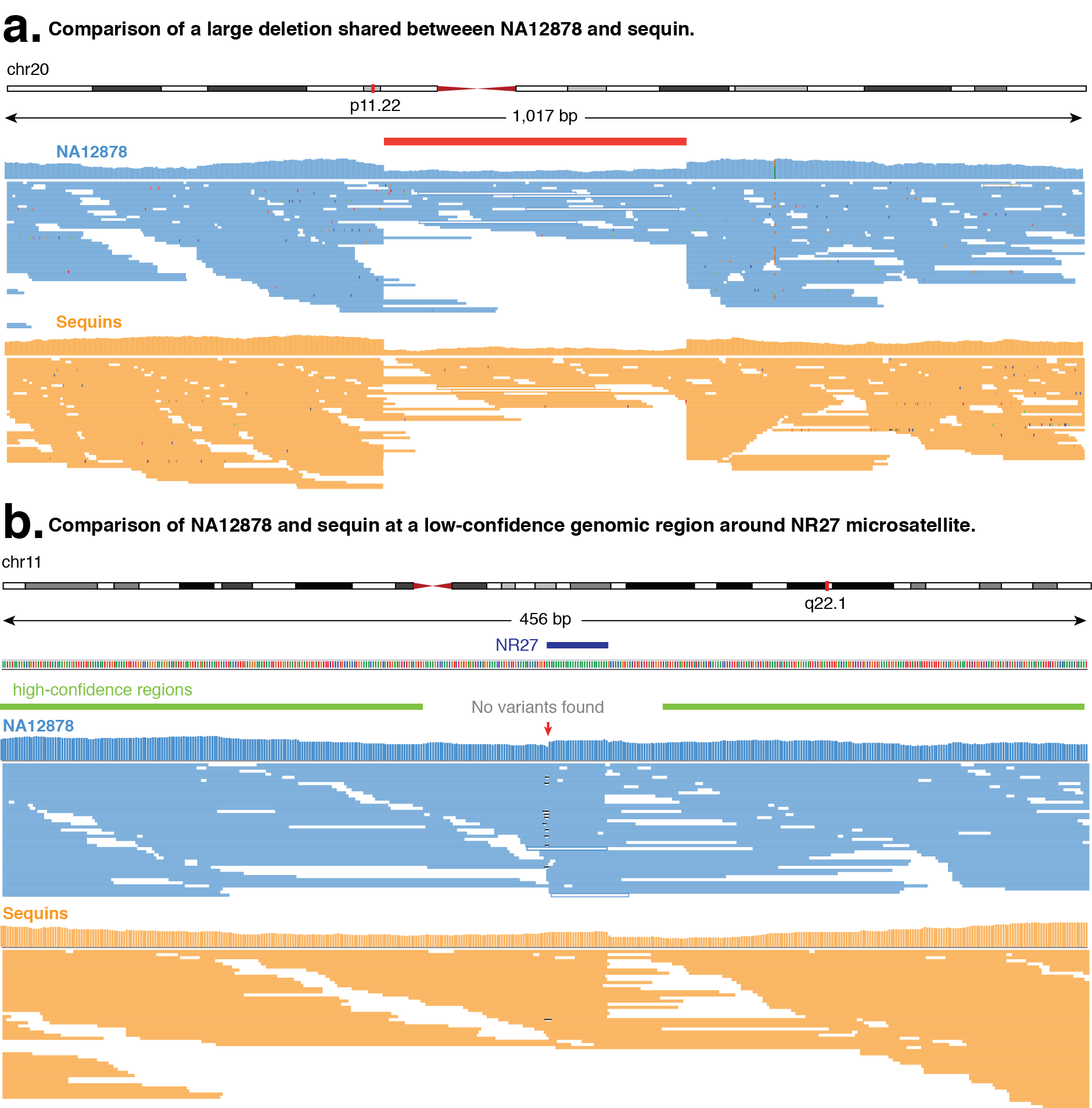


**Fig. S2. Commutability between NA12878 and sequins. (a)** Alignment of NA12878 and sequin reads (HiSeqXTen/PCR-free) at a large heterozygous deletion present in NA12878 and also represented by sequins. Sequins can faithfully emulate well-characterised genetic variation. **(b)** Alignment of NA12878 and sequin reads (HiSeqXTen/PCR-free) at a microsatellite (NR27, blue bar), not covered by the GIAB NA12878 high-confidence regions (green bar). Even though there are deletions in the reads consistent with a pattern of heterozygous variation (red arrow), variation in low-confidence regions is not included in reference annotation. A comparison with sequin aligments for the same region would make it easier to distinguish between bona fide variation and sequencing errors. **(a-b)** For each library NA12878 data is represented in blue and corresponding sequin data is represented in orange.

**
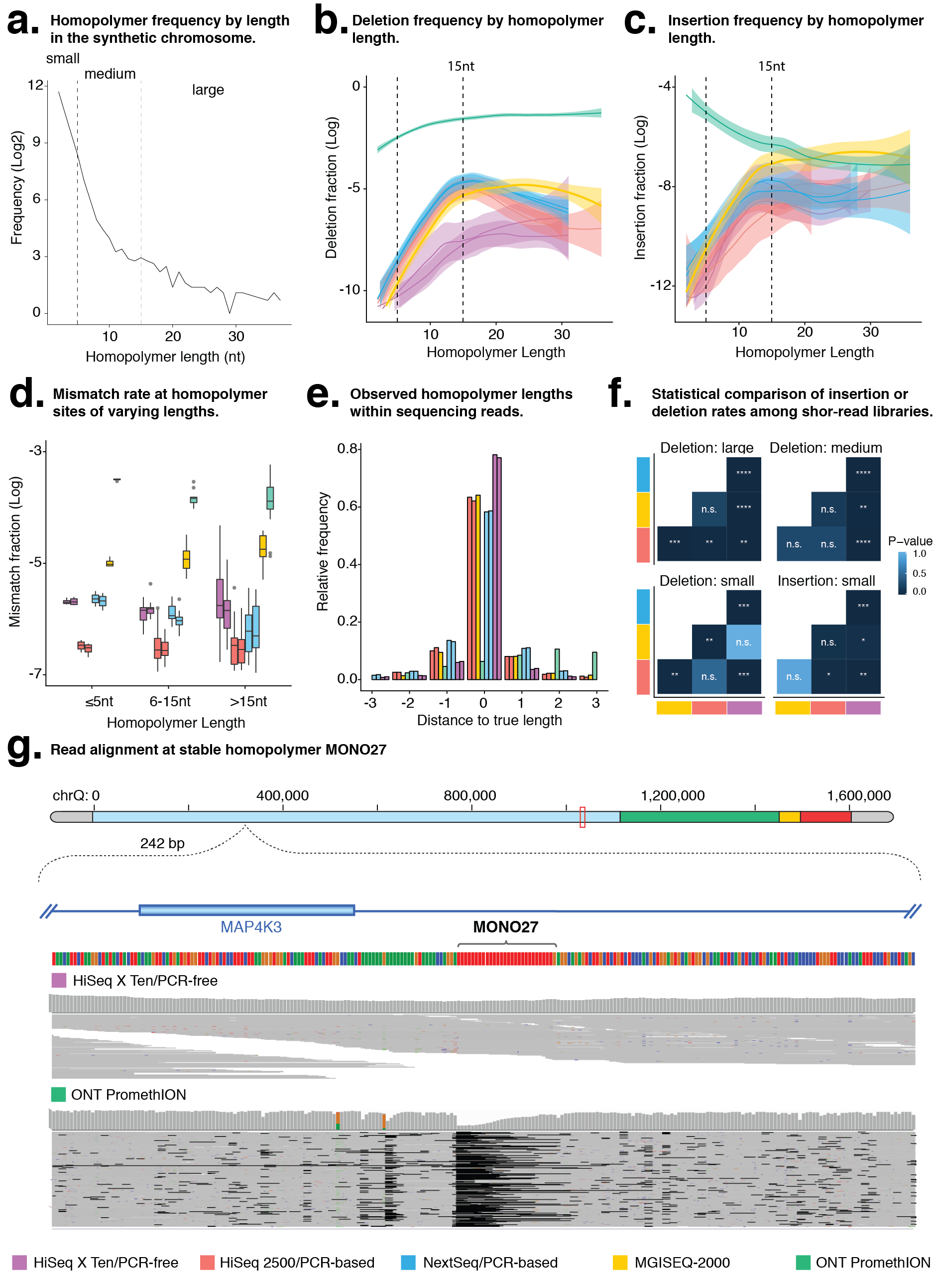
**

**(See Over) Fig. S3. Performance at simple repeats. (a)** Absolute frequency of all homopolymer sequences of varying lengths within the synthetic chromosome (small=178,799; medium=1,918; large=105). The dashed vertical lines demarcate the different length categories (small ≤ 5nt, medium > 5nt & ≤ 15nt, large > 15nt). **(b-c)** Regression lines showing the relationship between homopolymer length and the frequency of deletions and insertions, respectively. Overall the frequency of deletions and insertions increase with homopolymer length up to 15nt and then stabilizes. **(d)** Frequency of mismatches at small (≤ 5nt), medium (> 5nt and ≤ 15nt) and large (> 15nt) homopolymers. **(e)** Relative frequency of the distance between observed versus the true length of homopolymers for all reads spanning large homopolymers. **(f)** Pairwise Mann-Whitney-Wilcoxon test between short-read libraries for the frequency of deletions and insertions within homopolymers of different lengths (small ≤ 5nt, medium >5nt & ≤15nt and large > 15nt). P-values (p) were adjusted for a false discovery rate (FDR) of 0.05; p ≤ 0.05 (*), p ≤ 0.01 (**), p ≤ 0.001 (***), p ≤ 0.0001 (****), n.s.=non-significant. **(g)** Read alignment, for HiSeqXTen/PCR-free and ONT PromethION, at the representation of the stable homopolymer MONO27, present within an intron of the gene MAP4K3, in-between exons 13 and 14, in chromosome 2. The individual tracks consist of a histogram showing the depth of coverage at each position, in the top, and the aligned reads, in the bottom. The nucleotides in the reference sequence are coloured as follows, T=red, A=blue, C=green and G=orange, while individual mismatches in the reads are represented with those same colours. The black segments in the ONT reads represent indels. **(a-g)** The colours represent the different sequencing technologies/preparation methods, such as, HiSeq X Ten/PCR-free (purple), HiSeq 2500/PCR-based (red), NextSeq500/PCR-based (blue), MGISEQ-2000 (yellow) and ONT PromethION (green).

**
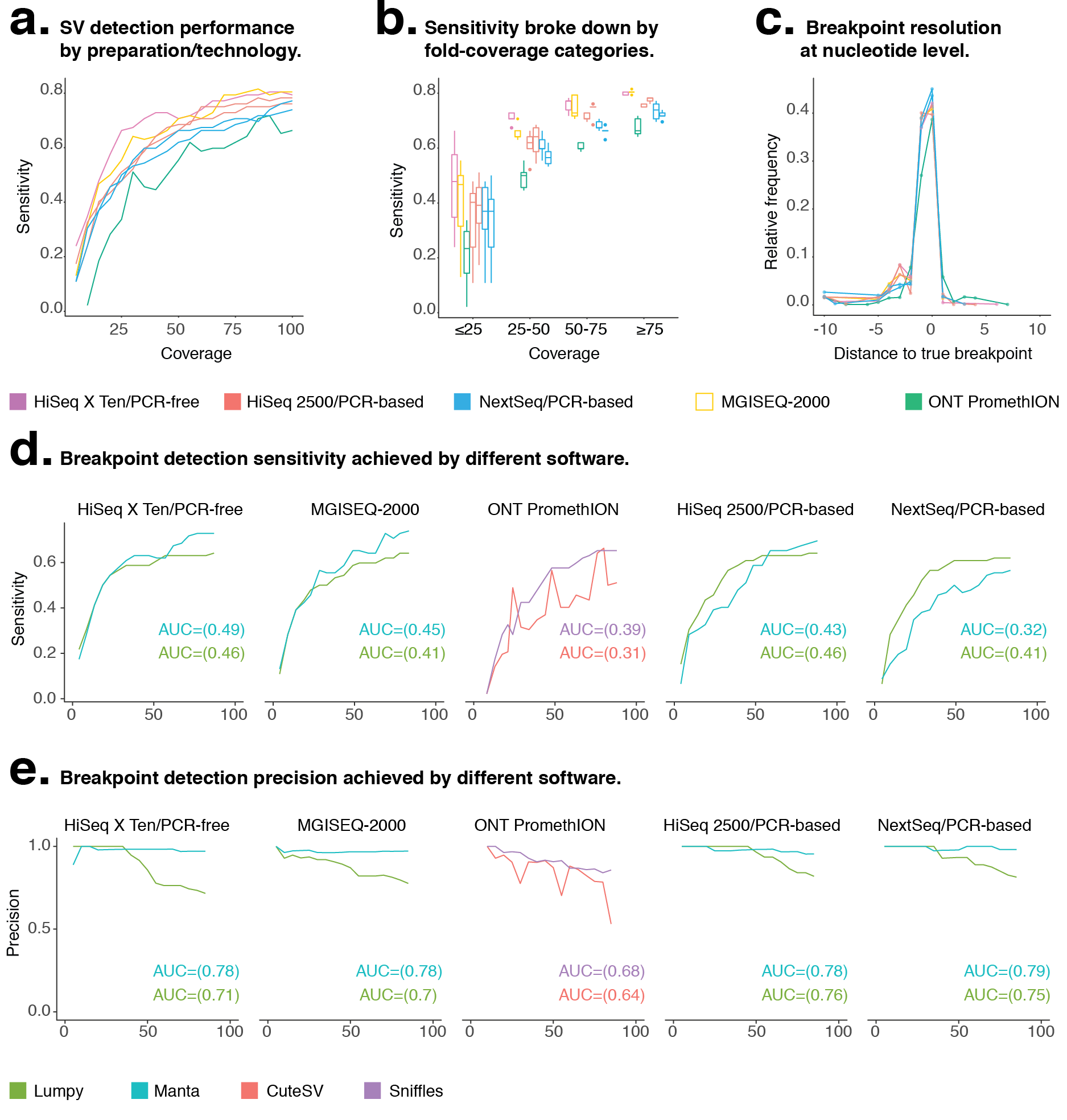
**

**Fig. S4. SV calling performance. (a)** Sensitivity of breakpoint detection for the different sequencing technologies/preparation methods, by aggregating results from different SV callers, at varying depths of coverage. **(b)** Nucleotide-level accuracy of detected breakpoints, by calculating the relative frequency of the distance between the observed and the true breakpoint positions. **(c)** SV calling sensitivity at the breakpoint level for short-read libraries (called with *Lumpy* or *Manta*) and long-read library (called with *NanoSV* or *Sniffles*) relative to sequencing coverage. **(d)** Sensitivity broken down by SV caller for the different technologies/preparation methods, with the area under the curve (AUC) indicated. **(e)** Sensitivity broken down by SV caller for the different technologies/preparation methods, with the area under the curve (AUC) indicated. **(a-c)** The colours represent the different sequencing technologies/preparation methods, such as, HiSeq X Ten/PCR-free (purple), HiSeq 2500/PCR-based (red), NextSeq500/PCR-based (blue), MGISEQ-2000 (yellow) and ONT PromethION (green). **(d-e)** The colours represent the different SV callers used, such as Lumpy (green), Manta (red), NanoSV (blue) and Sniffles (purple).

**
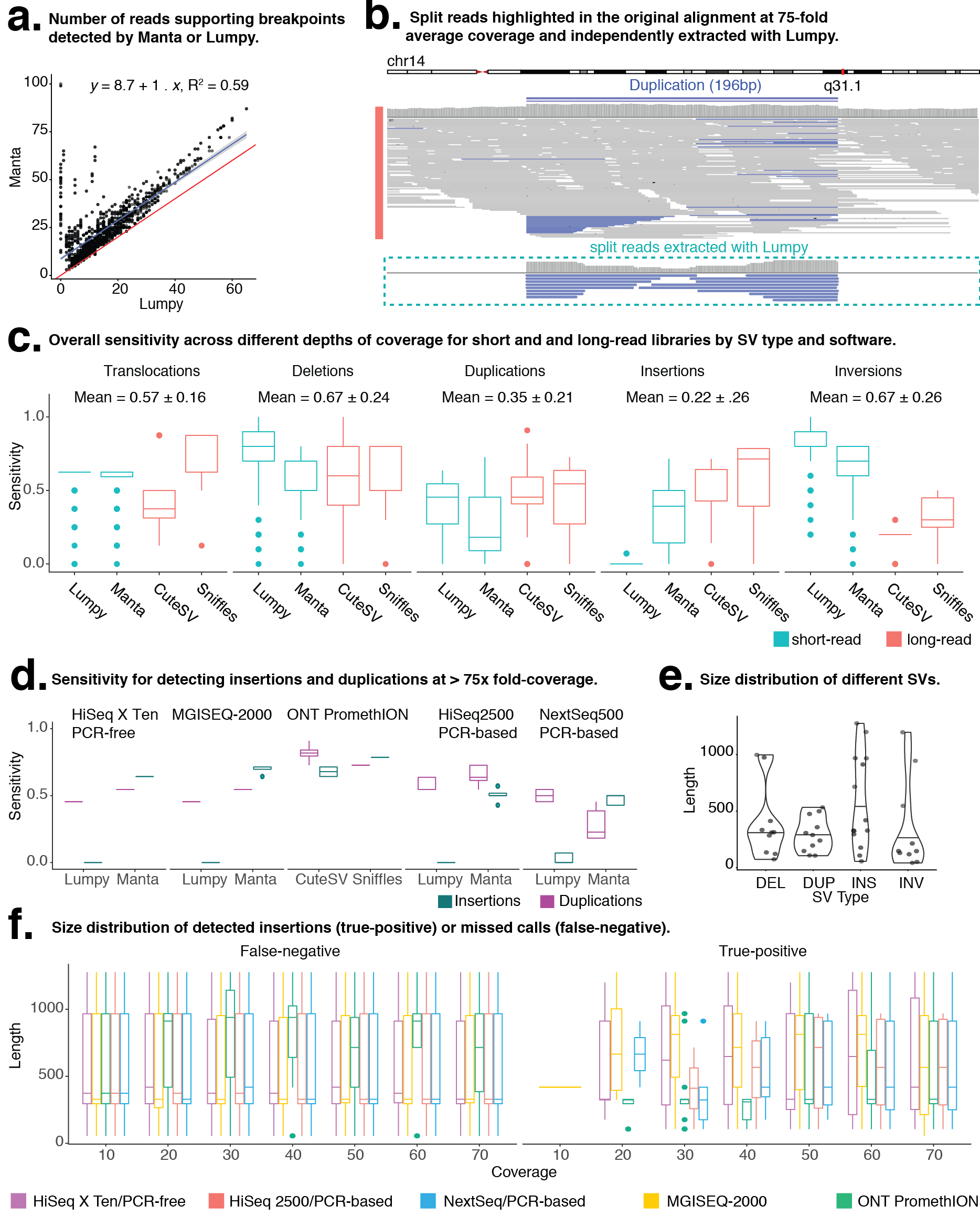
**

**(See Over) Fig. S5. Visualising structural variants. (a)** Number of supporting reads for breakpoints detected both with Lumpy and Manta. The regression line is represented in blue with its function and also R-squared indicated, whilst the identity line is represented in red. **(b)** Read alignment for HiSeq 2500/PCR-based library at segmental duplication (in blue). Split reads were highlighted in blue in the original alignment and also independently extracted with a script provided by Lumpy (cyan box). The number of supporting split-reads independently extracted was smaller than the number of split-reads tagged by the read aligner. **(c)** Distribution of the overall sensitivity obtained from short-read (green) and long-read (red) libraries across varying depths of coverage, using different SV callers, for each SV type. The mean sensitivity and standard deviation for each SV type is indicated. **(d)** Distribution of the sensitivity for detecting insertions and duplications obtained for different SV callers across the different technologies at depth of coverage higher than 75-fold. **(e)** Size distribution of different SV types represented in the synthetic chromosome (DEL=deletion, DUP=duplication, INS=insertion and INV=inversion). **(f)** Size distribution for true-positive and false-negative insertions at varying depths of coverage. **(a-f)** The colors represent the different sequencing technologies/preparation methods, such as, HiSeq X Ten/PCR-free (purple), HiSeq 2500/PCR-based (red), NextSeq500/PCR-based (blue), MGISEQ-2000 (yellow) and ONT PromethION (green).

**
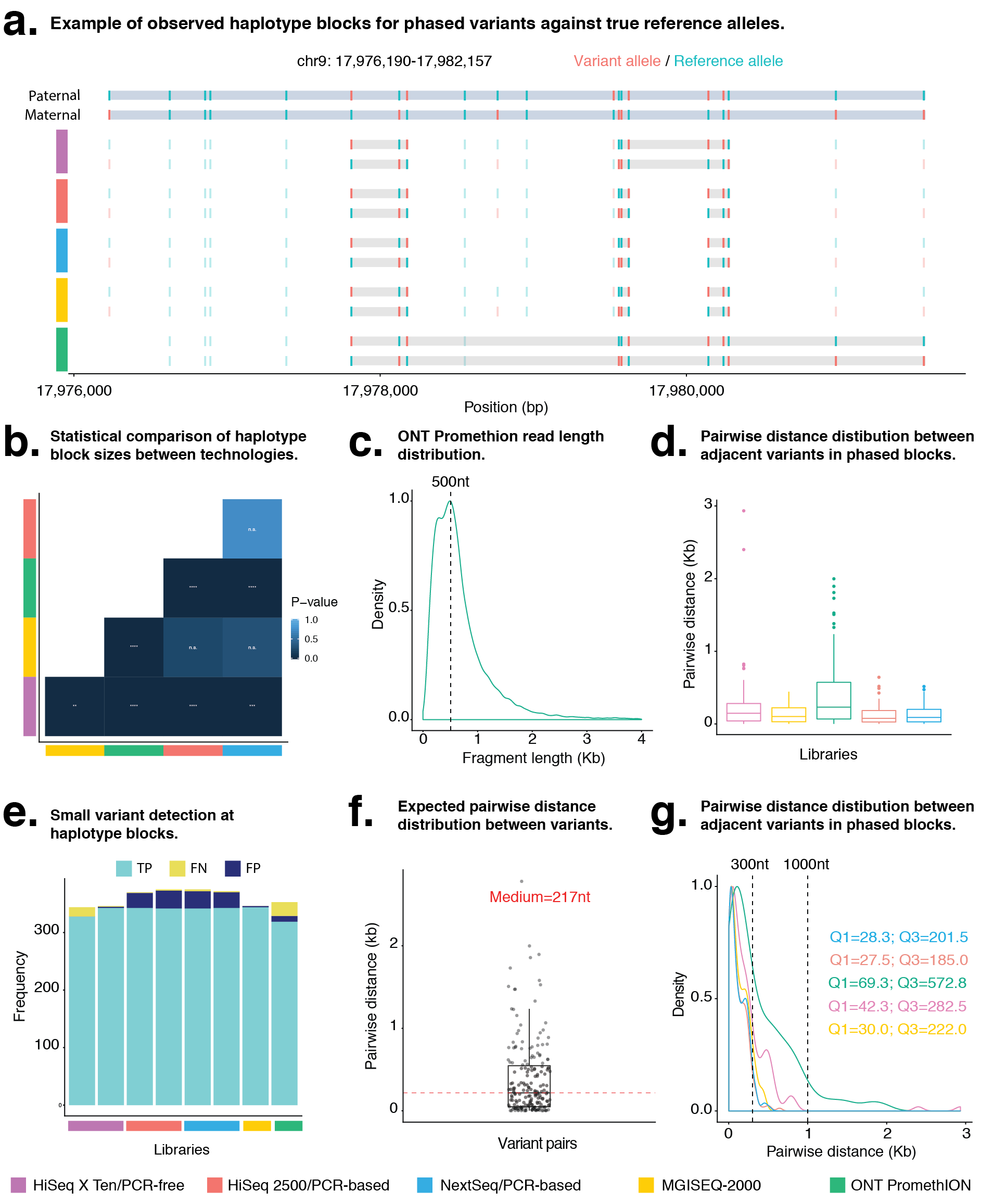
**

**(See Over) Fig. S6. Phasing performance. (a)** True haplotype blocks for a sequin representing chr9:17,976,190-17,982,157, showing reference (green) and variant (orange) alleles, and the actual haplotype blocks called in the different libraries. **(b)** Pairwise Mann-Whitney-Wilcoxon test between the different technologies for the size of assembled haplotypes. P-values were adjusted for a false discovery rate (FDR) of 0.05; p ≤ 0.05 (*), p ≤ 0.01 (**), p ≤ 0.001 (***), p ≤ 0.0001 (****), n.s.=non-significant. **(c)** Read length distribution for ONT PromethION. **(d)** Observed pairwise distance distribution for heterozygous variants within the assembled haplotype blocks in the different technologies. **(e)** Frequency of variants detected at haplotype blocks, classified as false-negatives (beige), true-positive (light blue) and false-positive (dark blue). **(f)** Distribution of expected pairwise distance between heterozygous variants in the sequin haplotype blocks. The red dashed line represents the medium pairwise distance at 217nt. **(g)** Observed pairwise distance densities for heterozygous variants within the assembled haplotype blocks in the different technologies. The first and third quartiles of each distribution are indicated and the dashed lines represent 300 and 1000nt distance, respectively. **(a-g)** The colors represent the different sequencing technologies/preparation methods, such as, HiSeq X Ten/PCR-free (purple), HiSeq 2500/PCR-based (red), NextSeq500/PCR-based (blue), MGISEQ-2000 (yellow) and ONT PromethION (green).


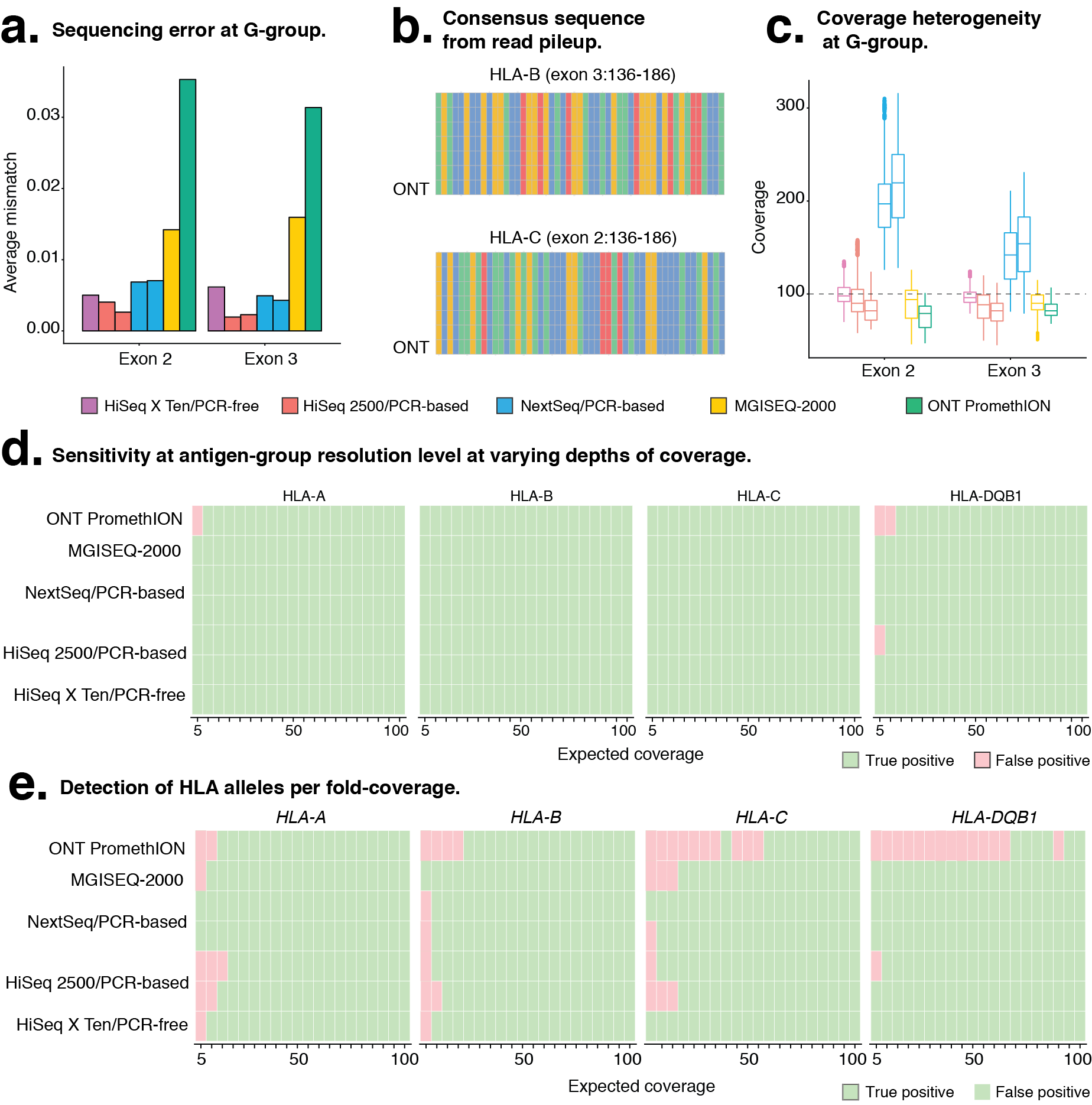


**Fig. S7. HLA typing performance. (a)** Observed sequencing error rate at exons 2 and 3 (G-group) in the different HLA loci represented in the synthetic chromosome. **(b)** Multiple sequence alignment comparing of the consensus sequence for exons 2 and 3 among the different sequencing libraries reveals no mismatches at HLA-B:exon3 and HLA-C:exon2. The nucleotides are coloured as follows T=red, A=blue, C=green and G=yellow. **(c)** The boxplots show the coverage distribution at individual positions for exons 2 and 3 (G-group) in the different HLA loci represented in the synthetic chromosome **(d)** Detection of HLA loci at the antigen resolution level relative to sequencing coverage in the different sequencing libraries. The green colour represents true-positive detections while red represents false-positives. **(e)** Detection of HLA loci at the allele resolution label relative to sequencing coverage in the different sequencing libraries. The green colour represents true-positive detections while red represents false-positives. **(a-e)** The colours represent the different sequencing technologies/preparation methods, such as, HiSeq X Ten/PCR-free (purple), HiSeq 2500/PCR-based (red), NextSeq500/PCR-based (blue), MGISEQ-2000 (yellow) and ONT PromethION (green).

**
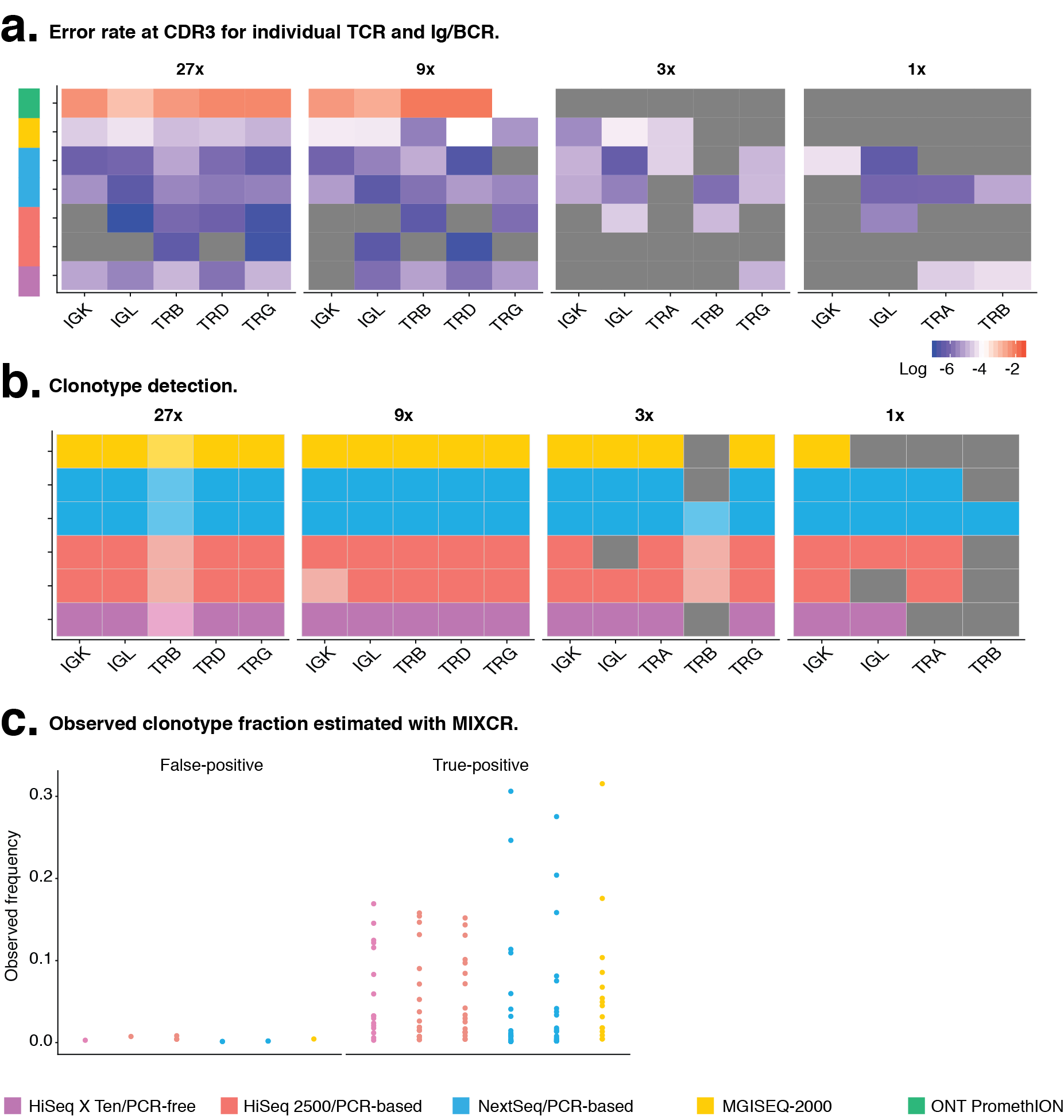
**

**Fig. S8. Immune repertoire analysis.** **(a)** Sequencing error at CDR3 region for different rearranged TCRs and BCRs. The colour gradient varies between blue to red, where full blue is the lowest error, the midpoint is white, and full red is equal to highest error rate. **(b)** Detection of TCR and BCR clonotypes at different expected frequencies (1-fold, 3-fold, 9-fold and 27-fold) in the different libraries. Full colour respective to each library represents CDR3 sequences correctly identified, whereas opaque colour means that the CDR3 sequence was correctly identified, but not the V(D)J segments and grey represents false-negatives. **(c)** Observed fractions estimated by MIXCR for false-positive and true-positive clonotypes.  **(a-c)** The colours represent the different sequencing technologies/preparation methods, such as, HiSeq X Ten/PCR-free (purple), HiSeq 2500/PCR-based (red), NextSeq500/PCR-based (blue), MGISEQ-2000 (yellow) and ONT PromethION (green).
