# Supplementary material for: Using synthetic chromosome controls to evaluate the sequencing of difficult regions within the human genome
Source: Genome Biol. 2022 Jan 12;23:19. doi: 10.1186/s13059-021-02579-6 (PMC8753822; doi:10.1186/s13059-021-02579-6)
Supplement: Supplementary file 9 — Additional file 9. Review history. [file 13059_2021_2579_MOESM9_ESM.docx]

Review History

**First round of review**

**Reviewer 1**

**Are you able to assess all statistics in the manuscript, including the appropriateness of statistical tests used?**

Yes, and I have assessed the statistics in my report.

**Comments to author:**

Dear authors,

This manuscript presents a well-written and impressive comparison of sequencing technologies on synthetic DNA fragments. It is however not the first manuscript to compare sequencing platforms. With high-quality genome assemblies available, it is imperative to state in which cases synthetic DNA fragments are an advantage and in which cases they are not an accurate representation. I do understand though that the current study was initiated well before the current state of high-quality haploid and diploid assemblies. For more general elements like structural variants, the manuscript does not improve on our current benchmarks. I would, however, put those things that are uniquely suited to a synthetic fragment benchmark more in the spotlight compared to e.g. structural variants, which would be more accurately tested using the full genome assemblies. The manuscript as currently written aims to be a general comparison of technologies, but I would suggest that a more specialized comparison on somatic variants, HLA, TCR, and BCR would be more impactful.

Please find my specific comments below,

Sincerely,

Wouter De Coster

Major comments

I worry that the synthetic fragments are not an accurate representation of long-read sequencing, as input DNA fragments for an actual (human) genome will most often be at least an order of magnitude longer. The fragment lengths in figure S6C are remarkable, but also the actual genomic DNA library seems rather short and could be suboptimally prepared, which could be majorly improved by current experience. The manuscript mentions both LSK108 and LSK109 for the ONT library prep. In addition, real DNA has methylation marks, which will be detected by the nanopore sequencing and are not present in synthetic fragments, or maybe are present in a bacterial context. And likely at least as important, Albacore v2.2.7 was released in April 2018 and is now, 3.5 years later, absolutely not representative of the current accuracy of nanopore basecalling. Albacore has been deprecated in favor of Guppy, and it has been shown repeatedly that repeating the basecalling with a more recent version substantially improves the results. These elements together make the inclusion of ONT in this comparison misleading and not a helpful description of the ability of the technology. Synthetic fragments seem highly unfit for benchmarking of long-read sequencing. These limitations warrant removing ONT sequencing from the manuscript or clearly stating that the performance as indicated is not as what would be expected for current actual genome sequencing.

Minor comments

I think a less ambiguous terminology could be 'virtual chromosome' rather than decoy or in silico chromosome.

I do not understand the choice for a diverging scale (blue-white-red) in Figure 3c and 4b, where only numbers between 0 and 1 have to be represented, and different shades of a single color would be a more natural representation.

In figure 1, panel B the authors mention only NanoSV and not Sniffles in the analysis tools, while I would argue that Sniffles is the current standard tool for long read SV calling. In contrast, NanoSV is not actively maintained and has been shown by comparisons to be not as accurate as Sniffles. Furthermore, WhatsHap is misspelled in the same panel.

The manuscript mentions the edge effect, and how this is mitigated. Is this effect also visible in the ONT data, or was the mitigation applied regardless?

The manuscript mentions both LSK108 and LSK109 for the ONT library prep.

The last two panels of Figure 2a (transition and transversion errors) may be more clearly represented by stacked bar charts of absolute numbers. It seems now that the ONT PromethION has fewer transversion errors than the other technologies, but this just seems to be the case because it has more transition errors.

**Authors Response**

**Point-by-point responses to the reviewers’ comments:**

This manuscript presents a well-written and impressive comparison of sequencing technologies on synthetic DNA fragments. It is however not the first manuscript to compare sequencing platforms. With high-quality genome assemblies available, it is imperative to state in which cases synthetic DNA fragments are an advantage and in which cases they are not an accurate representation. I do understand though that the current study was initiated well before the current state of high-quality haploid and diploid assemblies. For more general elements like structural variants, the manuscript does not improve on our current benchmarks. I would, however, put those things that are uniquely suited to a synthetic fragment benchmark more in the spotlight compared to e.g. structural variants, which would be more accurately tested using the full genome assemblies. The manuscript as currently written aims to be a general comparison of technologies, but I would suggest that a more specialized comparison on somatic variants, HLA, TCR, and BCR would be more impactful.

*We thank the review for their time and constructive feedback. We agree that ongoing improvements to assembly and sequencing are enabling an increasing number and characterisation of reference cell lines and genome materials. However, despite these advantages, we believe that synthetic controls provide numerous complementary benefits that should not be discounted.*

*In particular, synthetic controls is that they enable to ground-truth representation of complex and pathogenic mutations that are not otherwise present in reference cell-line genome materials. For example, the synthetic controls discussed within this manuscript provide representation of complex structural variants, viral insertions, immune receptors and oncogenic translations for which there are no available reference materials. Furthermore, reference cell lines also only harbor a small number of pathogenic mutations, whilst synthetic materials can harbor large numbers of mutations at precise allelic frequencies.*

*Similarly, whilst we agree that some of the benefits of synthetic controls are less applicable to long-read sequencing, we highlight that these technologies are not the sole focus of the manuscript, and we also performed benchmarking of short-read sequencing technologies that are used in the vast majority of clinical sequencing applications. Nevertheless, we wish to retain the long-read sequencing data and analysis throughout the manuscript provide a useful comparator to consider the relative advantages of current short- and long-read sequencing technologies.*

Major comments

I worry that the synthetic fragments are not an accurate representation of long-read sequencing, as input DNA fragments for an actual (human) genome will most often be at least an order of magnitude longer. The fragment lengths in figure S6C are remarkable, but also the actual genomic DNA library seems rather short and could be suboptimally prepared, which could be majorly improved by current experience. The manuscript mentions both LSK108 and LSK109 for the ONT library prep. In addition, real DNA has methylation marks, which will be detected by the nanopore sequencing and are not present in synthetic fragments, or maybe are present in a bacterial context. And likely at least as important, Albacore v2.2.7 was released in April 2018 and is now, 3.5 years later, absolutely not representative of the current accuracy of nanopore basecalling. Albacore has been deprecated in favor of Guppy, and it has been shown repeatedly that repeating the basecalling with a more recent version substantially improves the results.

*We thank the reviewer, and agree that a more updated version of nanopore sequencing and basecalling software is needed for a fairer representation of performance. Accordingly, as required we have updated the analysis, results and figures within the manuscript using the LSK110 kit (1D ligation), base-calling was performed using ONT Guppy Software (version 4.5.3). and ONT reads with minimap2 (version 2.22-r1105-dirty). Please see Materials and Methods for further details.*

These elements together make the inclusion of ONT in this comparison misleading and not a helpful description of the ability of the technology. Synthetic fragments seem highly unfit for benchmarking of long-read sequencing. These limitations warrant removing ONT sequencing from the manuscript or clearly stating that the performance as indicated is not as what would be expected for current actual genome sequencing.

*Whilst we understand the reviewer’s concerns, we disagree that synthetic fragments are highly unfit for long-read sequencing. Whilst there are some limitations due to fragment size, we do not agree that this discount the benefits of synthetic fragments, and believe they should be considered complementary to human reference materials. As indicated above, synthetic controls provide ground-truth examples of many clinically relevant mutations for which there are currently no other available natural reference standard materials. Accordingly, we collaborate with many clinical laboratories that have requested and use our synthetic controls to verify Oxford Nanopore sequencing - this clearly demonstrates the utility of synthetic controls for nanopore sequencing.*

*Given we have updated the data according to current best practises, we would prefer to retain the ONT analysis which to be an important contribution to our manuscript. In addition to providing a useful comparator to the short-read sequencing technologies, our data has provided tangible insights into the benefits and limitations of nanopore sequencing in clinical diagnosis. Nevertheless, we do agree with the reviewers’ concerns and have added the following qualifying statement within the discussion:*

*“Furthermore, the full advantages of long-read sequencing may not be fully realised in our benchmarking analysis given that synthetic controls used in this study are shorter than the typical read length achieved with long-read sequencing.”*

Minor comments

I think a less ambiguous terminology could be 'virtual chromosome' rather than decoy or in silico chromosome.

*The decoy chromosome was originally chosen because it is consistent with established terminology to describe the inclusion of foreign chromosome sequences in a reference genome assembly used to align foreign or viral sequences. For example, see https://www.cureffi.org/2013/02/01/the-decoy-genome/ Therefore, whilst we appreciate the ambiguity of this term, we prefer not to use an alternative terminology not in current use.*

I do not understand the choice for a diverging scale (blue-white-red) in Figure 3c and 4b, where only numbers between 0 and 1 have to be represented, and different shades of a single color would be a more natural representation.

*The use of a blue-white-red scale was chosen because it provides broader spectrum across which to discriminate differences that can otherwise be challenging to resolve for heat-maps.*

In figure 1, panel B the authors mention only NanoSV and not Sniffles in the analysis tools, while I would argue that Sniffles is the current standard tool for long read SV calling. In contrast, NanoSV is not actively maintained and has been shown by comparisons to be not as accurate as Sniffles. Furthermore, WhatsHap is misspelled in the same panel.

*We thank the reviewer for identifying the WhatsHap speeling mistake, which has now been corrected. We have also removed NanoSV from the benchmarking analysis. We have included more recent structural variant caller, cutesv, alongside sniffles, to provide a comparator method. The main text and results have been modified to reflect this change.*

The manuscript mentions the edge effect, and how this is mitigated. Is this effect also visible in the ONT data, or was the mitigation applied regardless?

*Edge effects occur with the lower coverage of short-reads at the terminal regions, and to mitigate this, we exclude the terminal 500nt from analysis. However, edge-effects are not observed for ONT sequencing due to the long-read lengths.*

The last two panels of Figure 2a (transition and transversion errors) may be more clearly represented by stacked bar charts of absolute numbers. It seems now that the ONT PromethION has fewer transversion errors than the other technologies, but this just seems to be the case because it has more transition errors.

*The left panel in Figure 2a indicates the absolute number of errors, showing the higher error rate for ONT long-rad sequencing compared to short-read sequencing. The right panels how the relative proportion of transversion/transition errors for each sequencing technologies. Whlst we understand the reviewers concerns, it is not possible to present the ONT transversion/transition error rate in absolute terms without minimising the other technologies, which are then difficult to resolve. Therefore we have attempted to present the three panels are presented together so they are sufficiently resolved and interpreted collectively. To address the reviewer’s concerns, we have clarified in the figure legend as follows:*

*“Figure 2. Sequencing performance metrics using sequins. (a) (left panel) Per-base normalized coverage and absolute sequencing error distributions for different sequencing libraries. (central and right panel) The relative frequency of transitions and transversions mismatch errors.”*

**Second round of review**

**Reviewer 1**

Dear authors, I appreciate the updates and clarifications made with regard to my suggestions, and I definitely recognize that a synthetic set of clinically relevant variants has its merits. Despite my initial reservations, the current structure of the manuscript is defensible. With the mention that relatively short synthetic fragments may not be an accurate representation of long-read sequencing, I consider including the PromethION in the comparison suboptimal but acceptable. I want to congratulate the authors on this thorough and well-executed comparison and have no further comments. Sincerely, Wouter De Coster
